# Development of a Stable Electrolyte for Dendrite Suppression and High‐Performance Zn–I_2_ Redox Flow Battery

**DOI:** 10.1002/smll.202513846

**Published:** 2026-06-07

**Authors:** Aparnasree Moothedath, Mohanraj Madeshwaran, Anjana Puthanpurayil Jayarajan, Bal Sydulu Singu, Ulaganathan Mani, Samanth Kokkiligadda, Soong Ho Um

**Affiliations:** ^1^ Department of Chemistry Amrita School of Physical Sciences Coimbatore Amrita Vishwa Vidyapeetham Coimbatore India; ^2^ Functional Materials Laboratory Amrita School of Engineering Coimbatore Amrita Vishwa Vidyapeetham Coimbatore India; ^3^ Department of Physics Amrita School of Physical Sciences Coimbatore Amrita Vishwa Vidyapeetham Coimbatore India; ^4^ School of Chemical Engineering Sungkyunkwan University Suwon Republic of Korea; ^5^ Department of Chemistry Sreenidhi University Hyderabad India; ^6^ SKKU Advanced Institute of Nanotechnology (SAINT) Sungkyunkwan University Suwon Gyeonggi‐do Republic of Korea

**Keywords:** bifunctional electrolyte, dendrite suppression, long cycle life, polyhalide electrolyte, redox flow cell

## Abstract

The zinc–iodide redox flow battery has gained importance due to its low cost, non‐corrosive nature, high solubility, high energy density, and high redox potential. However, low iodine utilization, the shuttle effect, Zn dendrite growth, and dead Zn formation at the anode, as well as imbalanced reaction kinetics, prevent it from achieving long‐term cycling stability. To address these issues, this work improves the performance of the Zn–I_2_ flow cell by adopting the safe, low‐cost bifunctional ammonium chloride as a neutral supporting electrolyte. NH_4_Cl helps reduce I_2_ precipitation and polyiodide formation by forming an I_2_Cl^−^ complex. On the anolyte side, the electrolytes reduce the dendrite formation and increases stability. The ammonium chloride supporting electrolyte added on both sides, KI + NH_4_Cl (catholyte) || ZnCl_2_ + NH_4_Cl (anolyte) has shown promising stability over 1000 cycles at a current density of 30 mA cm^−2^, with a coulombic efficiency (CE) and energy efficiency of 93.83% and ~75%, further the flow cell also delivered a maximum power density of 74.8 mW cm^−2^, which is far better than the bare KI (catholyte) and ZnCl_2_ (anolyte) electrolyte‐based flow cell.

## Graphical abstract

An NH_4_Cl additive stabilizes Zn–I_2_ redox chemistry in aqueous Zn–I_2_ redox flow batteries by suppressing iodine precipitation and mitigating zinc dendrite formation. These improve reversibility and electrolyte stability, leading to enhanced coulombic and energy efficiencies, as well as substantially prolonged cycling performance.

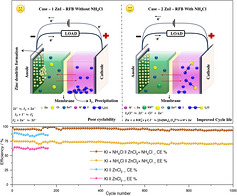

## 1 Introduction

In recent years, the demand for renewable energy has increased due to its advantages, such as low cost, cleanliness, and environmental friendliness. However, effective utilization is questionable due to its fluctuating and intermittent nature [1, 2]. Energy Storage Systems (ESS), particularly electrochemical energy storage systems, can be a key to solving the problems [3]. Li‐ion batteries have been widely used for large‐scale energy storage because of their high energy density and long cycle life [4]. However, the high cost and flammable nature limited its practical application in remote areas or in off‐grid conditions [5]. To address the limitations, a system capable of storing high energy for off‐grid applications is necessary [6]. In this context, the redox flow battery (RFB) has emerged as a promising candidate for large‐scale energy storage, particularly for integrating renewable energy sources, owing to its decoupling, design flexibility, long cycle life, modularity, and high safety [7]. Unlike other batteries, RFB stores energy in the soluble redox couples present in the electrolyte, which are stored in tanks outside the cell stack. RFB consists of a pair of electrodes, usually carbon‐based felts, a catholyte, an anolyte, a membrane, a current collector, and a pump. The other significant advantage of a redox flow battery is its tunable energy and power density, which is made possible by its decoupling nature. The energy density of the RFB can be enhanced by increasing the electrolyte volume, and the power density by varying the electrode area or the number of cells in the battery. This can enable a megawatt capacity for large‐scale energy storage applications [8, 9]. Among all RFBs, aqueous electrolytes based RFBs are attracted more than the organic‐ electrolytes due to their high cell performance, eco‐friendly, nontoxic behavior, low maintenance, and safety [10]. The all‐vanadium redox flow battery (all‐VRFB) is the most common and has been commercialized due to the high stability of vanadium, which can be used on both the anolyte and catholyte sides, reducing cross‐contamination; its high electroactivity; and its high efficiency and long‐term durability [11]. However, on a large scale, it faces obstacles due to its high cost, low solubility, low energy density of 15–30 Wh L^−1^, and difficulty in handling the corrosive acid [12]. Therefore, it is necessary to develop low‐cost, high‐energy‐density flow batteries to meet the need for large‐scale energy storage [13]. A zinc‐based aqueous redox flow battery is considered one of the most promising options for this large‐scale technology due to the following advantages [11]. Zinc has a high specific capacity of 820 mAh g^−1^ and undergoes two electron transfers, which increase the energy density of the system [14]. High solubility in aqueous electrolytes, and the Zn/Zn^2+^ redox couple has a high negative potential of −0.76 V vs. SHE, compared to V^2+^/V^3+^ at −0.26 V vs. SHE. Additionally, it exhibits fast reaction kinetics; compared to other metals, it offers low cost, high safety, and abundant reserves on Earth [14]. So far, various Zn‐based RFBs have been reported, such as Zn–Br_2_ [15], Zn–Fe [16], Zn–V [17], Zn–Mn [18], Zn–I_2_ [19], and Zn–Ce [20]. Among these, Zn–Br_2_ is commercialized due to its high specific practical energy density of 55 Wh Kg^−1^, high cell voltage of 1.84 V, and long cycle life [21]. However, its development is limited by the formation of Br_2_ vapor, which is both toxic and corrosive in nature, as well as the slow kinetics of Br^−^/Br_2_ [22]. The Zn–Fe achieves an energy density in the range of 30–70 Wh L^−1^ and is low‐cost, but the instability of iron in the positive electrolyte and hydrolysis limit its long‐term stability [16]. Whereas Zn–Mn is attractive due to its energy density of 50–80 Wh L^−1^ and the environmentally benign manganese chemistry, its practical application is still hindered by species crossover, sluggish reaction kinetics, and electrolyte instability [18]. Similarly, the Zn–Cl_2_ RBF offers an energy density of 70–80 Wh L^−1^ due to its high cell voltage but faces challenges related to chlorine gas generation, corrosivity, and safety concerns [23]. Compared with other zinc‐based systems, Zinc–iodine RFB offers a high energy density of 160 Wh L^−1^.Unlike bromine and chlorine, iodine is considered one of the most promising halogens because it is non‐corrosive, highly soluble, exhibits fast I^−^/I_3_^−^ kinetics (0.536 V vs. SHE), has good reversibility, is abundant in seawater, and has low cost [24, 25]. As a result, the zinc–iodide redox flow battery (ZIRFB) emerged as a promising electrochemical energy storage system due to the merits of zinc–iodide, which provide high energy density, low cost, and a safer system compared to other RFBs [26]. Despite the advantages of ZIRFBs, their practical implementation remains hindered as it faces numerous challenges, including dendrite growth on the anode side, the formation of dead Zn, poor cyclability, the formation of insoluble I_2_ and polyiodide, which limits energy density, polarization, and capacity attenuation due to the shuttle effect of soluble iodine species [19]. These challenges can be addressed by modifying the electrode, electrolyte, and membrane. Electrolyte engineering through the incorporation of additives or complexing agents and the addition of supporting electrolytes is considered one of the most feasible and effective methods for improving system efficiency. Weng et al. [24] employed bromide ions as a complexing agent to stabilize free I_2_ and facilitate the formation of I_2_Br^−^ in the ZnI_2_ electrolyte, thereby enabling 60 cycles. Similarly, Mousavi et al. [27] demonstrated ZIRFB employing Cl^−^ as a complexing agent in an ammonium iodide electrolyte, which exhibited enhanced performance and good cycle life. Furthermore, Lu et al. [29] investigated the incorporation of the thiocyanate anion as a complexing agent to improve capacity, with cycling exceeding 80 cycles. Organic compounds, such as propylene carbonate and polyvinyl pyrrolidone, were added as complexing agents to enhance the capacity of iodine‐based RFBs [28]. In this work, a novel electrolyte combination with common supporting electrolytes on both sides is proposed to enhance flow cell performance by addressing the shortcomings. Here, potassium iodide (KI) and zinc chloride (ZnCl_2_) are chosen as the catholyte and anolyte, respectively, and ammonium chloride (NH_4_Cl) is used as a supporting electrolyte on both sides, which is a low‐cost and safe option [27]. Ammonium salt is a non‐metallic ion that helps to suppress the zinc dendrite formation at the anode. Additionally, it forms a complex with iodine (I_2_Cl^−^), which reduces I_2_ precipitation and increases the availability of I^−^, thereby enhancing electrolyte conductivity, as observed here [29]. The hydrolysis of zinc in the anolyte can be prevented because the solution is slightly acidic in nature [30]. Due to NH_4_^+^, the reaction kinetics of both I_3_^−^/I^−^ and Zn^2+^/Zn will be improved. When ammonium chloride is added, the crossover of the Zn^2+^ ions will also be inhibited [31]. ZIRFB can be more efficient than ZBRFB since the I^−^/I_2_Cl^−^ is an efficient species due to its high reversibility and corrosion‐free nature [32].

In this work, the electrochemical performance of the anolyte and catholytes, with and without a supporting electrolyte, was evaluated using a three‐electrode setup. The addition of ammonium chloride as a supporting electrolyte exhibits high current density, low onset potential, low peak‐to‐peak separation, and enhanced conductivity. The calculated diffusion coefficient of 23.72 × 10^−6^ and 53.12 × 10^−6^ cm^2^ s^−1^ for catholyte and 3.21 × 10^−6^ and 2.17 × 10^−6^ cm^2^ s^−1^ for anolyte is enhanced due to the addition of NH_4_Cl. The flow cells were also constructed and tested under different conditions. The flow cells have been subjected to Galvanostatic Charge–Discharge (GCD) and polarization analysis to study the rate performance, cyclability, and power density of the constructed flow battery. The bare cell (KI || ZnCl_2_) delivered a high overpotential of 398 mV at 30 mA cm^−2^, a maximum power density of 47.02 mW cm^−2^, and 160 cycles, whereas KI + NH_4_Cl || ZnCl_2_ + NH_4_Cl shows a low overpotential of 280 mV and achieved a maximum power density of 74.8 mW cm^−2^. Also, the cell cycled up to 1000 cycles with CE of 93.8% and EE of 70.52%. This drastic improvement is due to the influence of the NH_4_Cl in both the catholyte and anolyte. In the catholyte, it helps reduce I_2_ precipitation and polyiodide formation by forming a complex (I_2_Cl^−^). At the anode, uniform zinc deposition and reduced dendrite formation are attained due to the addition of ammonium chloride. Therefore, the addition of ammonium chloride helps improve the overall performance of the zinc–iodine redox flow battery.

## 2 Results and Discussion

The schematic representation of the Zn–I_2_ RFB is shown in Scheme S1. During charging, the iodide anions (I^−^) in the electrolyte are oxidized to insoluble iodine (I_2_) (Equation 1). This unstable iodine then interacts with the remaining iodide ions in the electrolyte to form a stable polyiodide (I_3_^−^). Upon further oxidation, only one‐third of the iodide is converted to iodine I_2,_ where there is no free iodide to stabilize the iodine. Therefore, utilization is reduced to two‐thirds of capacity, with one‐third used to stabilize the I_2_  formation (Equation 3) [33]. In the anolyte, the Zn^2+^ is reduced to zinc metal, as shown in Equation (4). The overall system will show a cell voltage of 1.29 V.

**During charging**

**Catholyte**

(1) $$2I^{-}⇋\;I_{2}+2e^{-\;}\;E^{0}=0.535\;V\;\mathrm{vs}.\;\mathrm{SHE}$$

(2) $$I_{2}+I^{-}⇋{\;I}_{3}^{-}$$

(3) $$I_{3}^{-}+2e^{-}⇋3I^{-}\;E^{0}=\;0.536\;V\;\mathrm{vs}.\;\mathrm{SHE}$$

**Anolyte**

(4) $$Zn^{2+}+\;2e^{-}⇋\mathrm{Zn}\;\;\;E^{0}=\;-0.76\;V\;\mathrm{vs}.\;\mathrm{SHE}$$

**Overall cell reaction**

(5) $$2I^{-}+Zn^{2+}⇋I_{2}+\mathrm{Zn}\;E^{0}=\;1.295\;V\;$$

**SCHEME 1:**
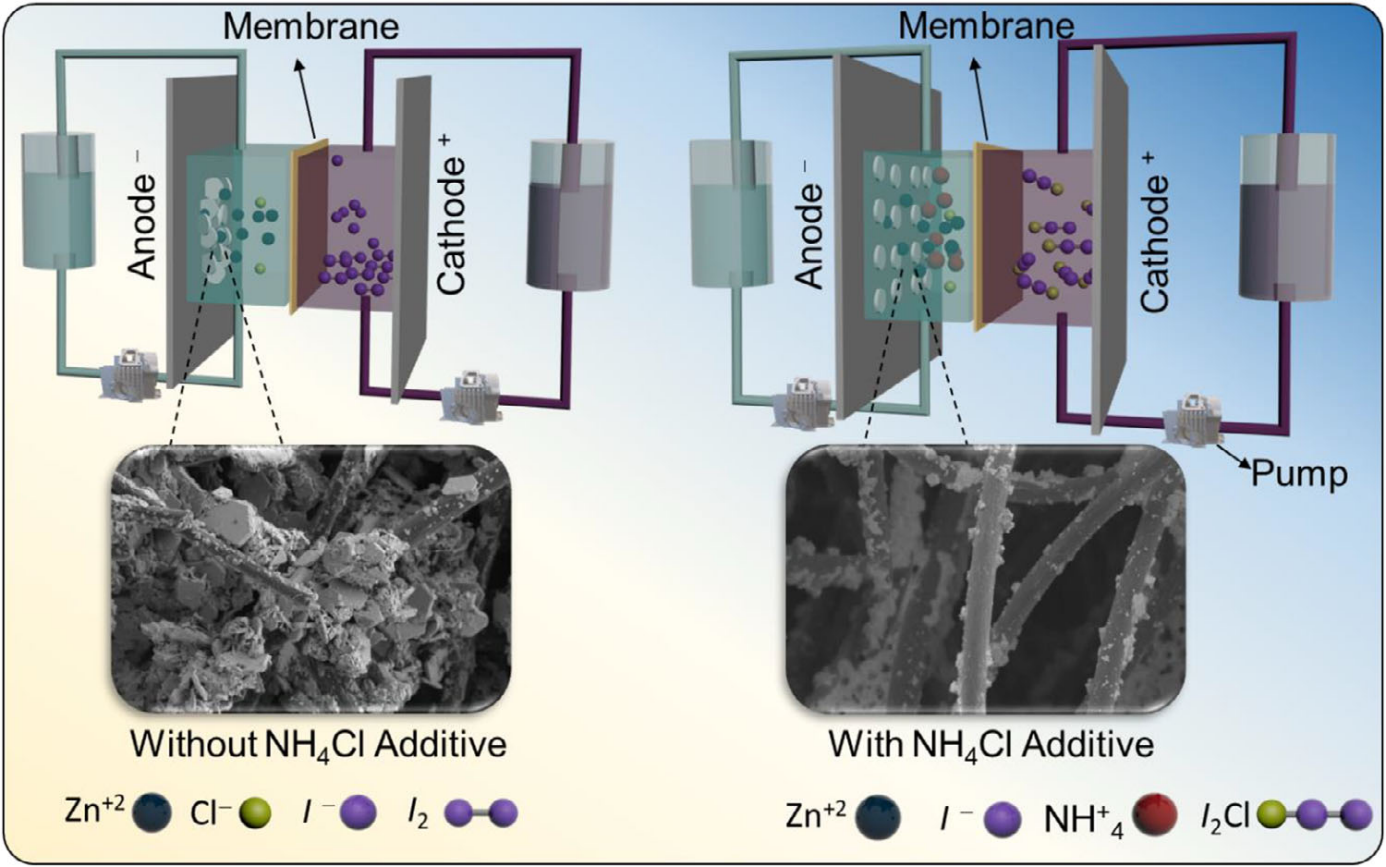
Schematic representation of the constructed redox flow battery. RFB without NH_4_Cl additive, and RFB with NH_4_Cl additive.

The difference between the normal Zn–I_2_ RFB and the supporting electrolyte‐added Zn–I_2_ RFB is shown in Scheme 1. In this work, the supporting electrolyte ammonium chloride exhibited dual behavior, providing both NH^4+^ and Cl^−^ counterions. Along with the Zn–I_2_ cell reactions, an intermediate chemical reaction occurs in which I_2_ is complexed with Cl^−^ to form the reversible I_2_Cl^−^. As a result, the utilization of I_2_ increases. On the other hand, in the anolyte, the incorporation of ammonium chloride forms a complex with the zinc, which helps to prevent the coupled formation of Zinc hydroxide. The addition of NH_4_Cl acts as a weakly acidic buffer, helping to prevent hydrolysis of zinc species in the electrolyte and providing uniform growth on the electrode [30].

During charging, as mentioned above, iodide is oxidized to iodine, which reacts with Cl^−^ in the electrolyte to form an I_2_Cl^−^ complex, thereby limiting the formation of I_2_ precipitate. Additionally, it unlocks or increases the iodide ions by freeing them from the iodide complex, forming an I_2_Cl^−^ complex, and enhances the performance of the zinc side. The NH_4_Cl will increase the kinetics of stripping and plating and also form a complex that further enhances performance. The overall cell voltage rises to 1.37 V vs. SHE due to the formation of the complex.

(6) $$I_{2}Cl^{-}⇋2I^{-}+\;Cl^{-}+\;2e^{-}\;E^{0}=\;0.61\;V\;\mathrm{vs}.\;\mathrm{SHE}$$

**Overall cell reaction**

(7) $$2I^{-}+Cl^{-}+Zn^{2+}⇋I_{2}Cl^{-}+\mathrm{Zn}\;E^{0}=\;1.37\;V\;\mathrm{vs}.\;\mathrm{SHE}$$

### 2.1 Half‐Cell Analysis of Catholyte

**FIGURE 1:**
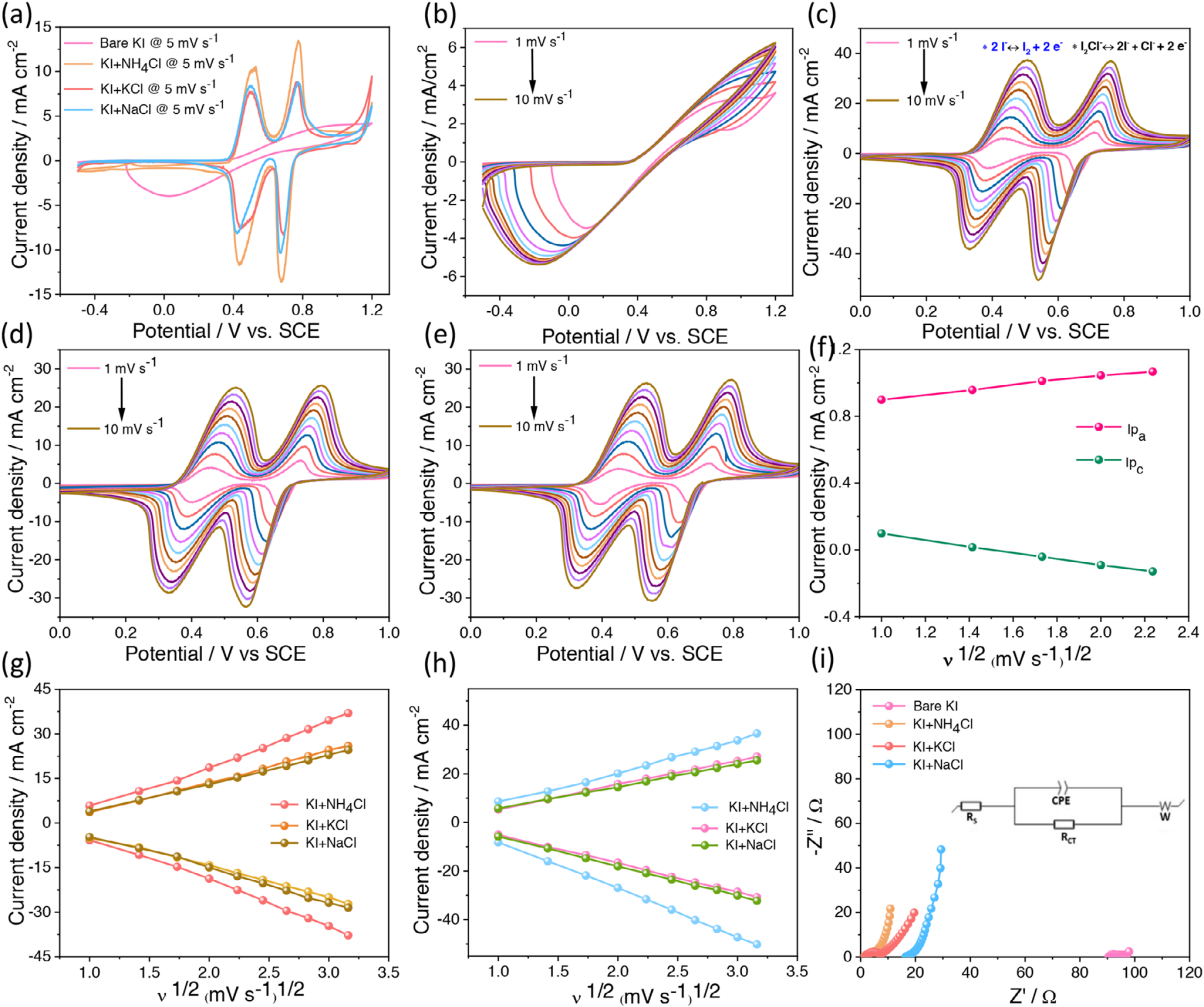
Electrochemical characterizations of catholyte: (a) shows the comparison of bare KI and supported electrolytes; added KI at 5 mV s^−1^ scan rate; (b) CV analysis of bare KI at different scan rates; (c) CV analysis of KI + NH_4_Cl at different scan rates. (d) CV of KI + KCl at different scan rates; (e) CV analysis of KI + NaCl at different scan rates; (d) Randle's Sevcik plot of bare KI;(e) Randle's Sevcik plot comparison of first peak of KI + NH_4_Cl, KI + KCl, and KI + NaCl;(f) Randle's Sevcik plot comparison of second peak of KI + NH_4_Cl, KI + KCl, and KI + NaCl; (i) Shows the EIS comparison of the bare KI and supporting electrolytes added KI from 100 kHz to 100 mHz frequency range with an amplitude of 10 mV.

To understand the effect of NH_4_Cl, both anolyte and catholyte with and without NH_4_Cl have been subjected to various electrochemical characterizations in three‐electrode cell testing. To elucidate the redox behavior of the I^−^/I_2_ couple, various supporting electrolytes were employed, and the best‐performing composition was used in the flow‐cell analysis. Here, three neutral pH‐supporting electrolytes have been selected, all of which share the common anion of chlorine (Cl^−^): KCl, NH_4_Cl, and NaCl, due to their high solubility and ionic conductivity. The supporting electrolytes used here are inert substances that enhance solution conductivity and are complexed with the counterions. As expected, incorporating supporting electrolytes reduces the internal resistance of the system, as evidenced by the redox flow cell analysis, which shows a lower IR drop [34]. Additionally, they help to control the pH and, hence, buffer the cell's capacity, thereby increasing the dissolution rate of counterions [35]. Figure 1a shows the CV comparison of the bare 0.01 m KI and 0.01 m KI + 1 m KCl, 0.01 m KI + 1 m NH_4_Cl, and 0.01 m KI + 1 m NaCl at the potential window ranging from −0.3 to 1.2 V vs. SCE. The bare KI shows a larger peak‐to‐peak separation potential of 800 mV compared to supporting electrolytes mixed with KI. Additionally, the current density for the supporting electrolyte‐added KI is superior to that of bare KI. Unlike KCl and NaCl, NH_4_Cl is a non‐metallic electrolyte with distinctive advantages. It undergoes hydrolysis in water, forming NH_4_^+^ and Cl^−^ ions that are safe, environmentally friendly, and have a sufficient lifespan in aqueous batteries [36]. Therefore, the supporting electrolyte added electrolytes exhibit low peak‐to‐peak separation and a lower onset potential. Additionally, compared to the bare KI, the solution with a supporting electrolyte shows well defined oxidation and reduction peaks. The first peak denotes the I^−^ to I_2_ conversion, whereas the second peak denotes the formation of the I_2_Cl^−^ complex. The supporting electrolyte helps prevent the formation of I_2_, which forms a passivation layer on the electrode, thereby reducing the electrode's kinetics [37]. Additionally, the stability and current density are enhanced by the addition of ammonium chloride and potassium iodide [38].

Figure 1b shows the CV of the bare KI electrolyte at different scan rates. It shows a high peak‐to‐peak separation and the formation of I_2_ and I_3_^−^. As the scan rate increases, the current density increases and shifts to a higher potential due to the higher overpotential associated with the electrolyte, or, in other words, the electrolyte's poor conductivity. It was observed that the CV does not exhibit a well‐defined peak during oxidation, indicating the electrochemical instability of the halide materials. In general, oxidation peaks are not observed in a CV curve due to the unstable nature of the species, particularly in aqueous solutions, which react readily with other species. If one formulates the electrolyte with appropriate complexing agents, this can prevent it, thereby improving cell performance [39]. Figure 1c shows the CV curves recorded for the KI + NH_4_Cl electrolyte at different scan rates at the potential window ranging from 0 to 1 V vs. SCE. From 0 to 1 V, the iodide to polyiodide and polyiodide to I_2_Cl^−^ complex takes place. Due to the presence of NH_4_Cl, an I_2_Cl^−^ complex is formed, which is absent in the bare KI. The bond between I_2_Cl^−^ is stronger than the I_2_I^−^ i.e., I_3_^−^ [40]. The precipitation of I_2_ is avoided, and polyiodide formation is limited, which improves the system's performance. The peak‐to‐peak separation is approximately 75 mV, indicating improved electrolyte conductivity [41]. On the other hand, the CV curves recorded at different scan rates for the electrolyte compositions KI + KCl and KI + NaCl are shown in Figure 1d,e. From the CV curves, the peak current density was determined for each scan rate at both the anodic and cathodic peaks; Figure 1f shows the square root of the scan rate vs. the current density plot for bare KI. The comparison of the supporting electrolyte, added KI, is shown in Figure 1g,h. Figure 1g shows the comparison of the iodide to iodine conversion, and Figure 1h shows the I_2_Cl^−^ complex conversion, respectively. The diffusion coefficient of the ions is calculated using Randles‐Sevcik's equation

(8) $$i_{p}=2.69\;\times \;10^{5}\times A\times C\times \nu^{1/2}\times \;n^{3/2}\times D^{1/2}\;\left(A\right)\;$$

where *i_p_* peak current density (A), A‐electrode surface area (cm^2^), C‐bulk concentration of the electrolyte (cm^−3^), ν‐scan rate (V s^−1^), n is the number of electrons transferred during the reaction, D‐diffusion coefficient (cm^2^ s^−1^). The diffusion coefficient obtained for the bare KI catholyte is 2.68 × 10^−9^ (anodic) and 4.42 × 10^−9^ cm^2^ s^−1^ (cathodic). On the other hand, the addition of NH_4_Cl to KI shows two oxidation and reduction peaks corresponding to the iodide‐to‐iodine conversion and the I_2_Cl^−^ complex. The diffusion coefficient values were estimated separately for them. The diffusion coefficient value for the iodide‐to‐iodine conversion is around 29.82 × 10^−6^ and 31.01 × 10^−6^ cm^2^ s^−1^ for the anodic and cathodic reactions, respectively, whereas the anodic and cathodic diffusion coefficient values for the I_2_Cl^−^ complex conversion are about 23.72 × 10^−6^ and 53.12 × 10^−6^ cm^2 ^s^−1^. The D values of KI + NH_4_Cl are high compared to those of the other supporting electrolyte added to the catholyte. Whereas, in the anolyte after adding the supporting electrolyte, the D values are reported to be 3.21 × 10^−6^ and 2.17 × 10^−6^ cm^2 ^s^−1^ for anodic and cathodic reactions, respectively, as given in Table S1. This improved behavior, along with the diffusion coefficient values, indicates enhanced kinetics of the redox reactions. To understand the electrode–electrolyte interfacial properties of the different electrolytes, EIS was recorded, and the obtained EIS data are compared in Figure 1i. KI + NH_4_Cl exhibits lower resistance values, R_s_ = 1.28 Ω and R_ct_ = 4.81 Ω, compared to the other electrolytes. Furthermore, the bare KI electrolytes exhibit high R_s_ (90.428 Ω) and R_ct_ (6.03 Ω). The exchange current density and the rate constant of the as‐prepared electrolytes, with graphite felt as the working electrode, have been estimated from the ESR values obtained. The observed R_s_, R_ct_, exchange current density, and rate constant values for all the prepared electrolyte compositions were compared in Table S2. KI + NH_4_Cl has high i_0_ (5.337 × 10^−3^ A cm^−2^) and K (5.530 × 10^−6^ cm s^−1^) values, indicating that, with this electrolyte combination, the electrode is more active and exhibits faster electron transfer [42]. The equation used to determine the electrode kinetic parameters, such as the rate constant and exchange current densities, is given in Equation S1a,b.

### 2.2 Half‐Cell Analysis: Anolyte

**FIGURE 2:**
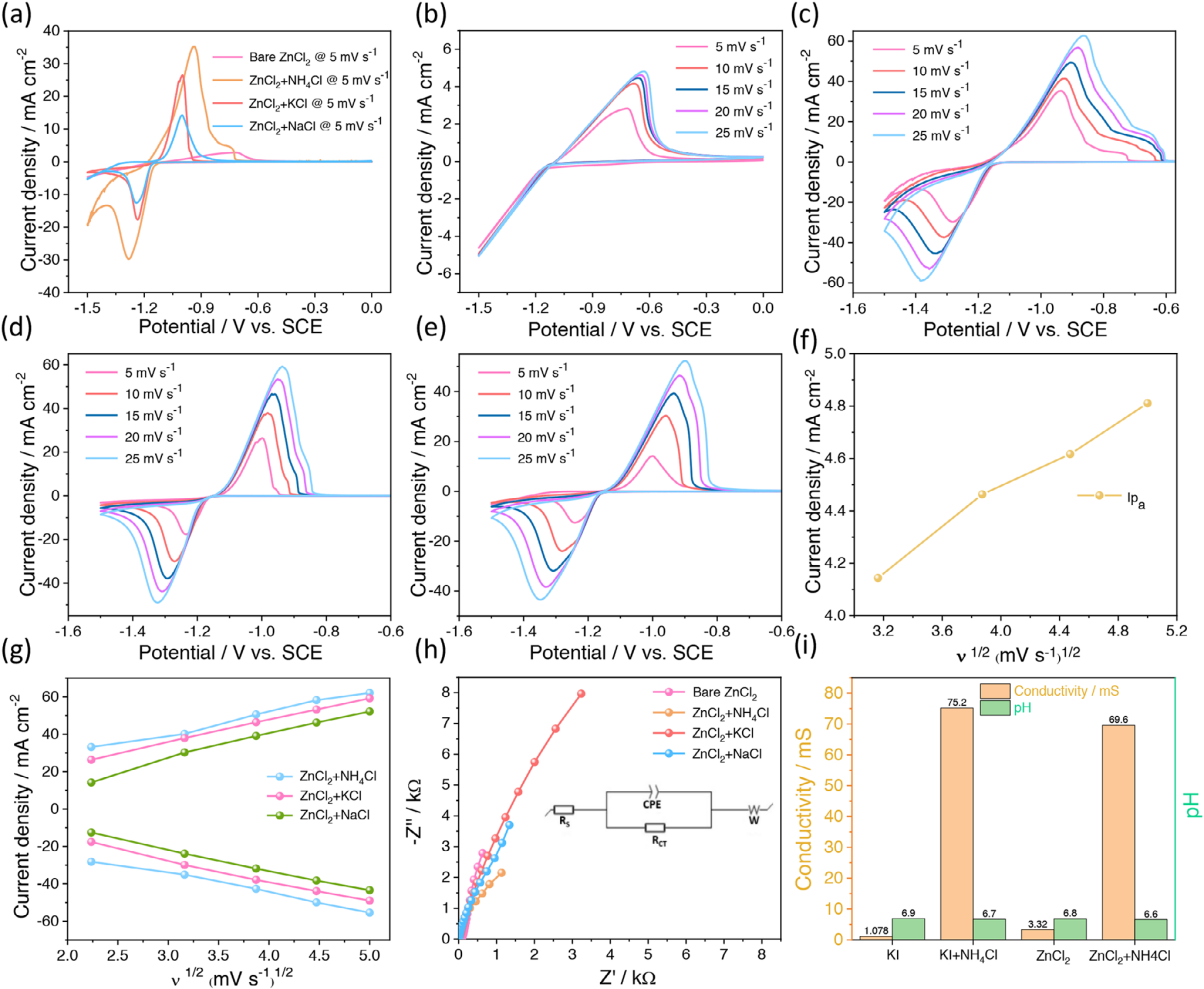
Electrochemical characterization of Anolyte: (a) comparison of bare ZnCl_2_, ZnCl_2_ + NH_4_Cl, ZnCl_2_ + KCl, ZnCl_2_ + NaCl at 5 mV s^−1^; (b) CV of bare ZnCl_2_ at different current scan rates; (c) CV of ZnCl_2_ + NH_4_Cl at different scan rates; (d) CV of ZnCl_2_ + KCl at different scan rates; (e) CV of ZnCl_2_ + NaCl at different scan rates; (f) Randle's Sevcik plot of bare ZnCl_2_;(g) Randle's Sevcik plot comparison ZnCl_2_ + NH_4_Cl, ZnCl_2_ + KCl and ZnCl_2_ + NaCl;(h) EIS comparison at 10 mV amplitude for the ZnCl_2_ and supported electrolyte added ZnCl_2_ in the range of 100 kHz to 100 mHz; (i) Conductivity and pH analysis of catholyte and anolyte.

Figure 2a shows the CV comparison of the bare ZnCl_2_, ZnCl_2_ + NH_4_Cl, ZnCl_2_ + KCl, ZnCl_2_ + NaCl, obtained at a 5 mV s^−1^ scan rate. The ZnCl_2_ + NH_4_Cl electrolyte exhibits a high current density of 35.17 mA cm^−2^, which is significantly higher than that of bare ZnCl_2_. Figure 2b shows the CV of bare ZnCl_2_ at the various scan rates of 5, 10, 15, 20, and 25 mV s^−1^, where the oxidation and reduction reactions on the curve clearly show the plating/stripping reaction of the Zn^2+^/Zn reactions. Figure 2c–e shows the CV of ZnCl_2_ + NH_4_Cl, ZnCl_2_ + KCl, and ZnCl_2_ + NaCl at different scan rates of 5, 10, 15, 20, and 25 mV s^−1^. Among the three supporting electrolytes, the combination of zinc chloride and ammonium chloride yields a higher current density than KCl or NaCl. Figure 2f shows the Randles‐Sevick plot for bare zinc chloride, from which the anodic diffusion coefficient has been calculated. Followed by the Randles‐Sevick plot for ZnCl_2_ + NH_4_Cl, ZnCl_2_ + KCl, and ZnCl_2_ + NaCl for anodic and cathodic peaks is shown in Figure 2g. From the Randles‐Sevick plot, the calculated diffusion coefficient values are shown in Table S1. Figure 2h shows the EIS of both electrolytes, from which it is clear that the bare ZnCl_2_ provides a higher series resistance than the ZnCl_2_ + NH_4_Cl. NH_4_Cl improves conductivity and facilitates uniform zinc deposition on the felt surface, while also suppressing zinc dendrite formation during cycling [43, 44, 45]. The ionic conductivity and pH of the electrolytes were measured using a Con 700 and pH 700 (Eutech instrument, Singapore), as shown in Figure 2i. The conductivity of a 0.01 m KI solution is approximately 1078 µS, and its pH is about 6.9, indicating that it is neutral. However, when 1 m NH_4_Cl is added, the conductivity increases to 75.2 mS, and the pH becomes slightly acidic due to the presence of ammonium chloride. On the anolyte side, ZnCl_2_ exhibits a conductivity of 3.32 mS and a pH of 6.8. When a supporting electrolyte is added, its conductivity increases to 69.6 mS, and the pH decreases slightly to 6.6, resulting in a slightly acidic solution.

### 2.3 Full Cell Analysis

**FIGURE 3:**
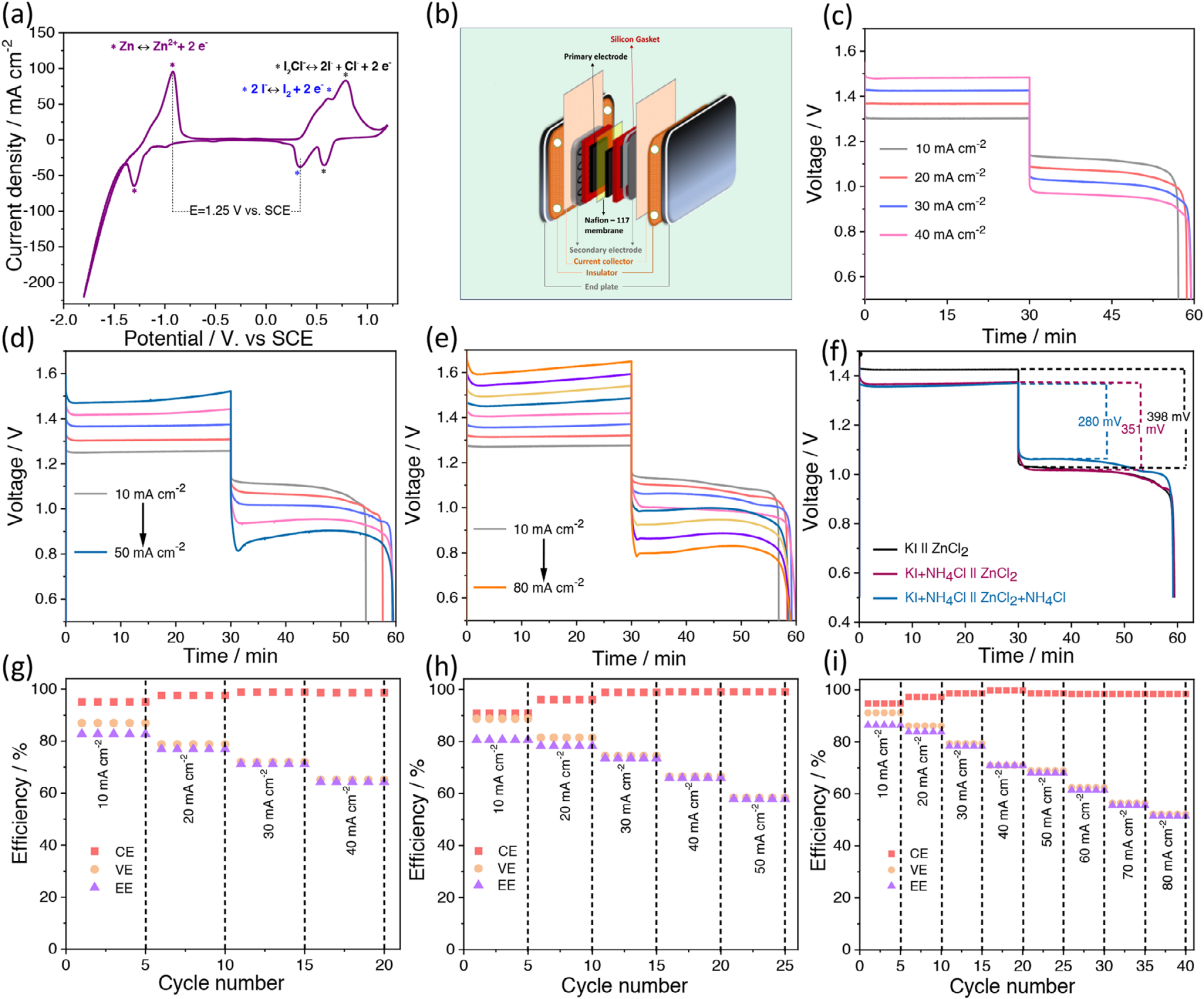
(a) Cyclic voltammetry of the 0.01 m KI + 0.01 m ZnCl_2_ + 1 MNH_4_Cl recorded at a scan rate of 5 mV s^−1^ at a potential window of −1.8 to 1.0 V vs SCE using a graphite felt; (b) Schematic representation of the cell components of the constructed flow cell; (c) Charge–discharge profile of bare KI || ZnCl_2_ at different current densities; (d) GCD of KI + NH_4_Cl || ZnCl_2_ cell at different current densities; (e) GCD of KI + NH_4_Cl || ZnCl_2_ + NH_4_Cl at different current densities; (f) GCD comparison of all three cells at 30 mA cm^−2^ current density. Efficiency comparison at different current densities: (g) Bare KI || ZnCl_2_; (h) KI + NH_4_Cl || ZnCl_2_; (i) KI + NH_4_Cl || ZnCl_2_ + NH_4_Cl.

The cyclic voltammetry analysis of iodide and zinc half cells has been carried out at a scan rate of 5 mV s^−1^ from the potential window ranging from −1.8 to 1.2 V vs. SCE using 0.01 m of zinc chloride(anolyte) and 0.01 m of potassium iodide(catholyte) with 1 m of ammonium chloride (supporting electrolyte). From the CV curve obtained, the total cell voltage was estimated to be 1.25 V vs. SCE, as shown in Figure 3a. The ZIRFB full cell is constructed using 1 m KI as the catholyte and 2 m ZnCl_2_ as the anolyte. The Graphite felt, consisting of a 2 × 2 cm^2^ area, is used as the active electrode, and Nafion‐117 is used as the membrane. A schematic representation of the cell components of the constructed flow cell is shown in Figure 3b. The photograph of the real‐time constructed flow cell setup is provided in the supporting information (Figure S2). The effect of NH_4_Cl on the flow cell for both anolyte and catholyte is studied. The cells were named as KI || ZnCl_2_, KI + NH_4_Cl || ZnCl_2_, KI + NH_4_Cl || ZnCl_2_ + NH_4_Cl. GCD analysis was performed for all these cells at different current densities to evaluate their performance. Figure 3c shows the GCD profiles of the KI || ZnCl_2_ cell recorded at different current densities ranging from 10 to 40 mA cm^−2^. At high current densities of 50 and 60 mA cm^−2^ (Figure S3e,f), there is a drastic decrease in discharge time due to poor mass transport at the electrode–electrolyte interface, low electrolyte conductivity, and electrolyte starvation. The First five cycles recorded at different current rates ranging from 10 to 40 mA cm^−2^ are shown in Figure S3a–d. To determine the influence of the NH_4_Cl supporting electrolyte on the catholyte, a flow cell has been constructed as 2 m KI + 1 m NH_4_Cl || 2 m ZnCl_2_, and the GCD analysis is shown in Figure 3d. The addition of NH_4_Cl significantly enhances the system's performance, increasing the current density to 50 mA cm^−^^2^. There is a drastic decrease in the IR drop. This improvement has been attributed to two key factors: the reduced precipitation of iodine (I_2_) and the formation of the I_2_Cl^−^ complex in the catholyte. Minimizing iodine precipitation maintains the availability of iodine in the reaction, while the I_2_Cl^−^ complex helps stabilize the iodine, preventing its loss from the system [32, 46]. Overall, the incorporation of NH_4_Cl optimizes electrochemical reactions by promoting efficient iodine utilization. At a high current density of 60 mA cm^−2^, the system is unstable due to problems on the anolyte side, as it has low conductivity, which makes the mass transport poor, as shown in Figure S4f. The continuous GCD profiles obtained at different current densities for the NH_4_Cl incorporated catholyte are shown in Figure S4a–e.

**FIGURE 4:**
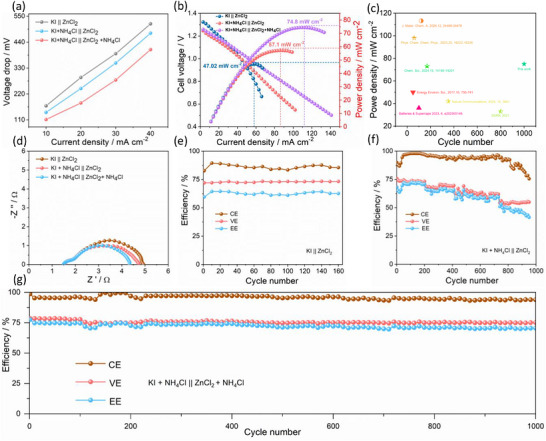
(a) Current density vs. Voltage drop profile of systems at 10–40 mA cm^−2^; (b) Polarization study comparison of Bare KI || ZnCl_2_; KI + NH_4_Cl || ZnCl_2_; KI + NH_4_Cl || ZnCl_2_ + NH_4_Cl; (c) Power density and cycle life comparison with existing literatures; (d) EIS analysis of the flow cell at 10 mV amplitude from 100 kHz to 100 mHz frequency range. Cycle life analysis of the constructed flow cell at 30 mA cm^−2^: (e) Bare KI || ZnCl_2_; (f) KI + NH_4_Cl || ZnCl_2_; (g) KI + NH_4_Cl || ZnCl_2_ + NH_4_Cl.

To inhibit the Zn dendrite formation at the anode, NH_4_Cl was added to both anolyte and catholyte, and the flow cell performance was investigated. GCD profile (third cycle) of the KI + NH_4_Cl || ZnCl_2_ + NH_4_Cl flow cell is compared at different current rates (Figure 3e). Notably, the system achieved a maximum current density of 80 mA cm^−2^ without significant adverse effects, including electrolyte crossover, electrolyte leakage, or pressure variations. This finding clearly demonstrates the pivotal role of NH_4_Cl as a supporting electrolyte, which appears to enhance the system's electrochemical performance. Specifically on the anolyte side, the addition of NH_4_Cl facilitates the formation of a complex with zinc chloride, effectively mitigating a critical issue in zinc‐based systems: dendrite formation [27, 47]. The formation of zinc dendrites leads to a reduction in the efficiency of the system. It compromises the safety of zinc‐based systems and represents a significant advancement toward more reliable and efficient zinc‐ion batteries [27, 48]. Further, the continuous first five GCD cycles recorded at different current densities are shown in supporting information Figure S5a–i. The GCD curves of the studied flow cells at a current density of 30 mA cm^−^^2^ are illustrated in Figure 3f. This comparison aims to assess the influence of adding a supporting electrolyte on the performance of ZIRFB, both with catholyte alone and with anolyte and catholyte together. All three systems demonstrated similar efficiency levels; however, there was a notable reduction in the internal resistance (IR) drop when NH_4_Cl was incorporated into both the anolyte and catholyte compartments. Specifically, the bare configuration exhibited a significant IR drop of 398 mV, primarily attributed to the passivation layer that formed on the catholyte side due to poor iodine conversion [49]. In contrast, the flow cell of KI + NH_4_Cl || ZnCl_2_ showed an improved performance with a reduced IR drop of 351 mV, which is better than that of a bare cell. Further, to enhance the overall efficiency and stability of the ZIRFB system. The IR drop for the KI + NH_4_Cl || ZnCl_2_ + NH_4_Cl flow cell significantly drops to 280 mV, which evidences the influence of the NH_4_Cl on both the catholyte and anolyte. Overall, it was observed that the IR drop of the KI + NH_4_Cl || ZnCl_2_ + NH_4_Cl cell is reduced to 30% when compared to the KI || ZnCl_2_ cell, where the cell electrode components remain the same. The Coulombic Efficiency (CE), Voltage Efficiency (VE), and Energy Efficiency (EE) of the constructed flow cells have been evaluated for each charge–discharge cycle at different current densities. The formula used to calculate the CE, VE, and EE has been given in the supporting information, Equation S2a–c. Figure 3g shows the efficiency profile of the bare KI || ZnCl_2_ cell at different current densities ranging from 10 to 40 mA cm^−2^. The improvement in coulombic efficiency resulting from increased current density is attributed to enhanced mass transport at the electrode–electrolyte interface. The CE, VE, and EE at 10, 20, 30, and 40 mA cm^−2^ are (95.16%, 86.96%, and 82.96%), (97.61%, 78.77%, and 76.88%), (98.83%, 72.02%, and 71.18%), and (98.63%, 65.16%, and 64.27) %, respectively. The efficiencies were calculated for KI + NH_4_Cl || ZnCl_2_ and KI + NH_4_Cl || ZnCl_2_ + NH_4_Cl cells to understand the effect of adding a supporting electrolyte. Figure 3h shows the efficiency of the KI + NH_4_Cl || ZnCl_2_ flow cell which gives CE, VE, and EE of (90.83%, 88.73%, and 80.6%), (96.03%, 81.47%, and 78.24%), (98.83%, 74.36%, and 73.49%), (99.1%, 66.53%, and 65.93%)%, and (99.1%, 58.35%, and 57.83%) at the current densities of 10, 20, 30, 40, and 50 mA cm^−2^. There is a slight improvement in the system's efficiency compared to the bare case, due to the influence of supporting electrolytes, which reduces the shuttle effect of the iodine species in the electrolyte [50]. The KI + NH_4_Cl || ZnCl_2_ + NH_4_Cl cell efficiencies were noted as (94.73%, 91.17%, and 86.37%), (97.26%, 86.13%, and 83.77%), (98.66%, 79.34%, and 78.28%)%, (99.83%, 70.98%, and 70.86%), (98.66%, 68.90%, and 67.98%), (98.46,% 62.40%, and 61.44%), (98.46%, 56.49%, and 55.62%), and (98.46%, 52.22%, and 51.42%) at 10 to 80 mA cm^−2^ are shown in Figure 3i. The efficiency comparison at 40 mA cm^−2^ is given in Figure S6a. There is a drastic increase in efficiency, attributed to the influence of supporting electrolytes on both the anolyte and catholyte sides. The zinc dendrite formation has been suppressed in the cell due to the ammonium ion forming a complex with zinc, [Zn(NH_3_)_x_Cl_y_]_2‐y_, which facilitates a favorable, uniform deposition of the electrode rather than uneven dendrite formation [51]. Additionally, on the catholyte side, the lower iodine utilization resulting from the formation of insoluble I_2_ during charge and discharge is a drawback of iodine‐based batteries, and this has been addressed by forming an I_2_Cl^−^ complex with an NH_4_Cl additive. The KI + NH_4_Cl || ZnCl_2_ + NH_4_Cl shows a low voltage drop of 404 mV at 40 mA cm^−2^, whereas the other two cells deliver 517.65 mV (KI || ZnCl_2_) and 478 mV (KI + NH_4_Cl || ZnCl_2_ + NH_4_Cl), respectively, as shown in Figure 4a. To elucidate the maximum power density of the Zn–I flow cells, a polarization study was conducted by charging the cell for 1 h at 40 mA cm^−2^ and then discharging at different current densities ranging from 3 to 140 mA cm^−2^, with a step of 3 mA every 5 s. Figure 4b shows the polarization curve for the fabricated flow cells. It was observed that the bare cell of KI || ZnCl_2_ showed a maximum power density of 47.02 mW cm^−2^ at 57 mA cm^−2^. The KI + NH_4_Cl || ZnCl_2_ maximum power density of 57.1 mW cm^−2^ at 87 mA cm^−2^. Thus, the additive incorporated catholyte can be operated at a higher current density than the bare system. Moreover, when NH_4_Cl is added to both sides of the KI + NH_4_Cl || ZnCl_2_ + NH_4_Cl cell, the power density increases to 74.8 mW cm^−2^ at 114 mA cm^−2^, which is well correlated with the GCD profile obtained here. From the comparison graph, it's noted that power density increases linearly with increasing current density, whereas cell voltage decreases. At 80 mA cm^−2^, KI + NH_4_Cl || ZnCl_2_ + NH_4_Cl delivers a specific capacity of 2.58 Ah L^−1^ with a corresponding energy density of 2.13 Wh L^−1^.The comparison of current density vs. Specific capacity of the KI + NH_4_Cl || ZnCl_2_ + NH_4_Cl cell is shown in Figure S6b.The capacity, specific capacity, energy density, and power density of the cells were calculated using the formulas given in the, Equation S3a–d. The flow cell with added supporting electrolyte yields better performance than the bare system. The availability of iodide species, as well as the uniform zinc deposition, improves with the addition of NH_4_Cl, a supporting electrolyte. Moreover, the power density and cycle life of the zinc iodide redox flow battery are compared with those reported in the literature. The reported power densities and cycle life are from Wang et al. SSRN 2021 (33 Wh kg^−1^, 800 cycles), Monalisa Chakraborty et al. [46] (36 Wh kg^−1^, 100 cycles), and Zhiquan Wei et al. [52] (42 Wh kg^−1^, 350 cycles), Guo‐Ming Weng et al. [24], (50 Wh kg^−1^_,_ 50 cycles), and Yichan Hu et al. [53] (73 Wh kg^−1^_,_ 170 cycles), Abena A. Williams (98 Wh kg^−1^, 60 cycles), and Ruhan Zhao [54] (113 Wh kg^−1^, 120 cycles) are shown in Figure 4c. The EIS of the flow cells recorded shows that the equivalent series resistance of both sides of the ammonium chloride flow cell is 2.76 Ω, which is less than that of the other cell systems, Figure 4d. The resistance values are shown in Table S3.

Cycle stability analysis is crucial for assessing the long‐term stability of a redox flow battery at a commercial scale. In this study, all three cells were subjected to long‐term charge–discharge analysis at a current density of 30 mA cm^−^^2^ for 15 min of charge and 15 min of discharge. Figure 4e–g shows the cycle life analysis of the constructed flow cell. The bare KI || ZnCl_2_ flow cell runs up to 160 cycles with 85.54% CE as shown in Figure 4e. After 160 cycles, the flow was stopped due to the large drop in efficiency. This was due to the formation of a zinc dendrite, the formation of dead Zn on the anolyte side, and, mainly, the limited concentration of iodide in the catholyte. Figure 4f presents the cycle life analysis of the KI + NH_4_Cl || ZnCl_2_ cell, indicating a CE of 75.6%. The VE and EE were very low at 54.92% and 41.52%, respectively, at the 950^th^ cycle. This is due to the heavy dendrite formation on the anolyte side, and this was again confirmed by Raman imaging (Figure S8b). The felt exhibits nonuniform dendrite growth, resulting in an increased pressure drop during cell operation. However, the obtained cycle life is significantly better than that of the bare electrolyte‐based flow cell, which was tested only up to 160 cycles. When NH_4_Cl is added to both the catholyte and the anolyte, the system's performance and stability improve markedly. The flow cell shows a cycle life of 1000, with CE of 93.88%, VE of 75.12%, and EE of 70.52% (Figure 4g). The high performance of the system is due to the formation of I_2_Cl^−^ and the Zn‐NH_4_Cl complex, which helps to reduce dendrite formation and increase the availability of iodide species in the electrolyte (Figure S8c). The cycle life is limited to 1000 cycles due to the small electrode area of 4 cm^2^ and the high flow rate of 70 mL min^−1^. The three cells were compared at the 150^th^ cycle, which shows that both sides of the NH_4_Cl‐added cell have higher efficiency than the other cells (Figure S6c). The comparison of each 100^th^ cycle from the cycle life is extracted and plotted in Figure S9a,b, which shows that both sides with added ammonium chloride exhibit a stable IR drop until the 1000th cycle. In contrast, the other cell having ammonium chloride (catholyte) shows a sharp voltage drop. The Zinc Iodide flow cell performance comparison of different works is given in Table S4.

### 2.4 Pre and Post Studies on the Effect of Supporting Electrolyte

**FIGURE 5:**
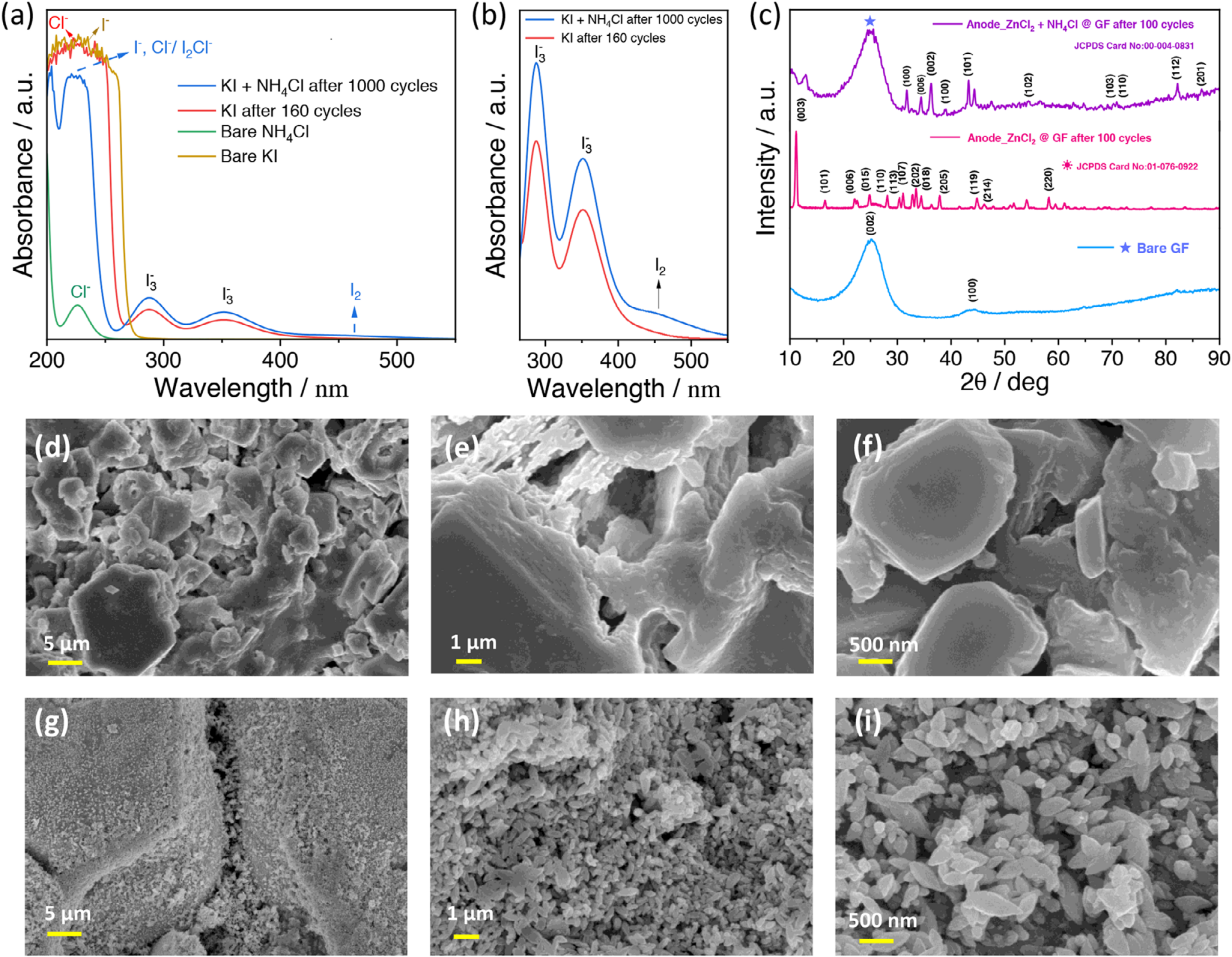
(a) UV–vis spectroscopy analysis of bare KI, bare NH_4_Cl and the electrolyte used in KI || ZnCl_2_ and KI + NH_4_Cl || ZnCl_2_ + NH_4_Cl after 160 and 1000 cycles, respectively; (b) Enlarged version of UV; (c) XRD of bare Graphite Felt (GF), anode of KI || ZnCl_2_ cell after 100 cycles, and anode of KI + NH_4_Cl || ZnCl_2_ + NH_4_Cl @GF electrodes after 100 cycles; (d–f) FE‐SEM analysis of anode of KI || ZnCl_2_ cell after 100 cycles; (g–i) FE‐SEM of anode of KI + NH_4_Cl || ZnCl_2_ + NH_4_Cl @GF electrodes after 100 cycles.

To confirm the formation of I_2_Cl^−^ complexes, UV–vis spectroscopy analysis has been carried out for bare KI, bare NH_4_Cl, and the electrolyte used in KI || ZnCl_2_ and KI + NH_4_Cl || ZnCl_2_ + NH_4_Cl after 160 and 1000 cycles, respectively. Figure 5a shows the presence of halogens (I^−^, Cl^−^) absorption peaks at 224 nm, triiodide ($I_{3}^{-}$) peak at 288 and at 352 nm [55]; the formation of triiodide and polyiodide is due to the cycling of the redox flow cell, whereas the bare KI and NH_4_Cl uncycled electrolyte doesn't show any peaks other than (I^−^, Cl^−^) peaks at 223 nm. The enlarged version of the UV spectrum in (Figure 5b) shows an absorption peak at 464 nm for KI + NH_4_Cl after 1000 cycles, suggesting the presence of I_2_, which can be further utilized in the further redox reaction, whereas the same peak is not present in bare KI after 160 cycles [56].

**FIGURE 6:**
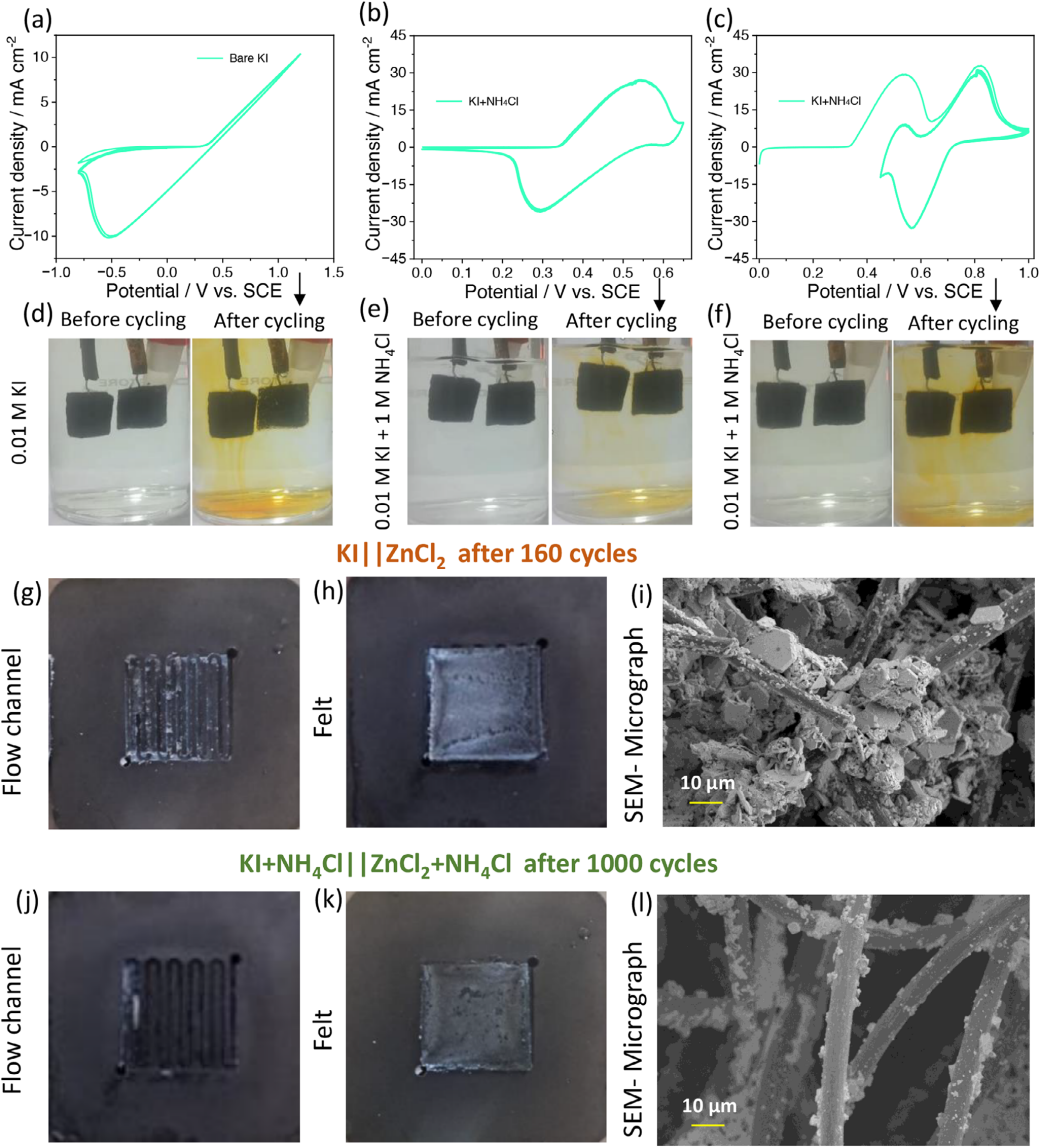
(a) Cyclic voltammetry of bare potassium iodide at a 10 mV s^−1^ scan rate; (b) shows the cyclic voltammetry of KI + NH_4_Cl at 10 mV s^−1^ at the potential window of ranging from 0–0.65 V vs. SCE; (c) shows the cyclic voltammetry of KI + NH_4_Cl at 10 mV s^−1^ at the potential window ranging from 0.44–1 V; (d–f) Photograph of before and after cycle of KI, KI + NH_4_Cl – peak 1, KI + NH_4_Cl – peak 2. Figure (g–i) shows post‐electrode analysis of the anode side of Bare KI || ZnCl_2_ after 160 galvanostatic charge–discharge cycles; Figure (j–l) shows post‐electrode analysis of the anode side of KI + NH_4_Cl || ZnCl_2_ + NH_4_Cl after 1000 galvanostatic charge–discharge cycles.

From this observation, the bare KI electrolyte, the 2I^−^ oxides to I_2,_ which is not stable, so it immediately reacts with I^−^ present in the electrolyte to form $I_{3}^{-}$(triiodide), which is poorly reversible due to low charge‐transfer kinetics. Also, it reduces the concentration of I_2_, which results in poor reverse reaction and reduces the concentration of I^−^, which is available for the next cycle. When the chloride‐containing electrolyte (NH_4_Cl) is added to the KI electrolyte after the first oxidation, the I_2_ product immediately bonds with Cl^−^ to form I_2_Cl^−^. I_2_Cl^−^ is also easily dissociable while the reaction is reversed, so no solid or semi‐solid product will be formed. Further, the I_2_Cl^−^ peak is present in the wavelength of 224 nm [57]. This is overlapped by the absorption peaks of halogens (I^−^, Cl^−^) [58]. To conclude, the addition of Cl^−^ to the KI electrolyte improves electrolyte reversibility, thereby increasing the cycle life of zinc iodine redox flow cells from 160 cycles to 1000 cycles. The morphology of zinc deposition and crystallographic orientation in systems with and without NH_4_Cl are characterized. The two cells of KI || ZnCl_2_, KI + NH_4_Cl || ZnCl_2_ + NH_4_Cl are constructed and cycled at 30 mA cm^−2^ current density for 100 cycles. Furthermore, the cell was dismantled while it was charged to study the morphology of zinc deposition and its crystallographic orientation. Figure 5c of the XRD shows the diffraction patterns of bare graphite felt, the anode of KI || ZnCl_2_ cell after 100 cycles, and the anode of KI + NH_4_Cl || ZnCl_2_ + NH_4_Cl @GF electrodes at 2θ of 10° to 90°. The bare graphite felt shows the (002) and (100) planes, which correspond to the crystalline peaks of graphite. The ZnCl_2_@GF anode after 100 cycles shows the formation of a by‐product, a zinc dendrite (zinc hydroxide chloride hydrate), with JCPDS card number 01‐076‐0922. Further, its morphology is shown in Figure 5d–f. Whereas the XRD pattern of the anode of KI + NH_4_Cl || ZnCl_2_ + NH_4_Cl @GF electrodes after 100 cycles clearly shows the deposition of metallic zinc which confirmed by the presence of the characteristic peaks (002), (100), and (101)(JCPDS card no. 00‐004‐0831). Additionally, the planes (100) and (006) correspond to ZnCl_2_, which is formed from the recrystallization of ZnCl_2_ electrolyte. The observation clearly shows that due to the addition of ammonium chloride, the zinc is deposited very uniformly. Moreover, the FE‐SEM images in Figure 5d–f show that zinc deposition without ammonium chloride results in a dense, irregular morphology made up of large platelet‐like agglomerates with relatively smooth surfaces. The zinc appears highly clustered with inconsistent grain growth, indicating uncontrolled nucleation and uneven deposition. These dense, coarse structures are often linked to localized zinc growth and could encourage dendrite formation during repeated cycling. Conversely, FE‐SEM images in Figure 5g–i reveal that adding ammonium chloride significantly changes the zinc deposition morphology. The zinc forms a more uniform, porous, and fine‐grained nanostructure composed of interconnected nanoscale flakes or particles. NH_4_Cl promotes uniform nucleation while suppressing excessive crystal growth, reducing particle size, and increasing surface roughness. This refined structure offers a larger active surface area and better ion diffusion pathways. The morphological change in the presence of NH_4_Cl suggests it effectively acts as an electrolyte additive to control zinc deposition kinetics, leading to more uniform plating and preventing dendritic or uneven growth. Such controlled deposition enhances the reversibility, cycling stability, and overall electrochemical performance of zinc‐based energy storage systems. Figure S7 shows the FTIR analysis of the cycle's anolytes. The bare ZnCl_2_ cycles at 160 cycles show peaks at 3529, 3053, 1617, 1433, and 919 cm^−1^. The peaks at 3529 and 3053 cm^−1^ correspond to O─H stretching of adsorbed water/hydroxyl. The interaction of the Zn─OH /O─H is shown by the peak at 1433, and the peak at 919 cm^−1^ assigns the stretching vibrations of the Zn─Cl / Zn─OH [59]. Figure S7 b shows the ZnCl_2_ + NH_4_Cl analysis. The presence of a new peak and shifts in peaks were observed. The peaks at 3152, 3016, and 2841 belong to the asymmetric stretching, symmetric stretching, and overtone combination of the N─H bond of NH^4+^. At 1270, the N─H wagging bond can be observed [60]. The FTIR spectra exhibited characteristic O─H and N─H vibrational bands associated with the hydrated ions and ammonium ions. Peak shifts and broadening of bonds were observed in ZnCl_2_ + NH_4_Cl, indicating interactions between the ammonium peaks and the Zn^2+^ solvation environment. Further, the effect of the supporting electrolyte (NH_4_Cl) on iodide reactions has been studied using an electrochemical half‐cell configuration. Figure 6a shows the cyclic voltammetry of bare potassium iodide at a 10 mV s^−1^ scan rate. The cyclic voltammogram of the KI electrolyte is characterized by a cathodic peak corresponding to the reduction of the electroactive iodine species, while the anodic peak corresponding to the reverse process does not appear. This suggests that the iodide/iodine redox couple, under the given experimental conditions, is electrochemically reversible. The overall process is described by the following steps: upon oxidation, I^−^ is first converted to I_2_ at the electrode surface; however, the formed I_2_ then rapidly undergoes a subsequent homogeneous chemical reaction with excess I^−^ in solution to form triiodide (I_3_^−^). Hence, this follows an EC‐type mechanism (electrochemical step followed by chemical conversion) and thus removes the oxidized species from the vicinity of the electrode before the reverse scan can re‐oxidize/reduce it, thereby suppressing the corresponding anodic peak. Further, the adsorption of iodine/polyiodide species on the electrode surface and their diffusion away from the interface may additionally result in reduced anodic features. Such kinetic and mass‐transport limitations are consistent with previous studies on halide electrochemistry, in which rapid chemical equilibria between I_2_ and I_3_^−^ give rise to apparent irreversibility in the voltammetric response.

Figure 6b shows the cyclic voltammetry of KI + NH_4_Cl at 10 mV s^−1^ at the potential window ranging from 0 to 0.65 V vs. SCE. The profile shows prominent oxidation and reduction peaks at 0.545 and 0.290 V vs. SCE, respectively. The observed peak is due to the conversion of 2I^−^ to I_2_. Further, the I_2_ immediately bonds with the Cl^−^ and forms I_2_Cl^−^, which prevents the poly‐iodide formation. Further oxidation of I_2_Cl^−^ is achieved by extending the potential window to 1 V. Figure 6c shows cyclic voltammetry of KI + NH_4_Cl at 10 mV s^−1^ in the potential window of 0.44–1 V. The oxidation and reduction peaks observed at 0.817 and 0.56 V vs. SCE are attributed to the oxidation of I_2_Cl^−^ to I_2_ and Cl^−^. Therefore, the three cyclic voltammograms obtained demonstrate the different complex reactions between iodide and chloride. The photographs of the as‐prepared and cycled electrolytes (cyclic voltammetry at 10 mV s^−1^ for three cycles) are shown in Figure 6d, which shows extensive polyiodide formation in the electrolyte solution, appearing yellowish‐brown. While the utilization of ammonium chloride suppresses the formation of polyiodide, the solution appears slightly yellow, mainly due to the formation of the I_2_Cl^−^ bond, as shown in Figure 6e. However, the slight formation of polyiodide is the rapid formation of I_2_ with unreacted I^−^, and this could be addressed by adding an excess of ammonium chloride. Moreover, the utilization of the I_2_Cl^−^ reaction, again, the I_2_ and Cl^−^ bond breaks, results in polyiodide formation, as shown in Figure 6f. The above observation clearly indicates that polyiodide formation can be effectively suppressed by adding ammonium chloride as a supporting electrolyte. The video recordings in Figure 6d–f are provided in supporting videos S1–S3. Figure 6g–i shows post‐electrode analysis of the anode side of Bare KI || ZnCl_2_ after 160 galvanostatic charge–discharge cycles. Figure 6g,h shows the huge dendrite formation at the graphite electrode channel and graphite felt due to the poor electrolyte conductivity and uneven deposition of the zinc while charging, which are clearly shown in the FE‐SEM image of the graphite felt in Figure 6i, whereas the KI + NH_4_Cl || ZnCl_2_ + NH_4_Cl cell shows very minimal zinc dendrite even after 1000 galvanostatic charge–discharge cycles due to the ammonium ion forming a complex with zinc, [Zn(NH_3_)_x_Cl_y_]_2‐y_, which facilitates a favorable, uniform deposition of the zinc on the graphite felt rather than uneven dendrite formation is shown in Figure 6j–l.

## 3 Conclusion

The influence of a supporting electrolyte on the ZIRFB is demonstrated using ammonium chloride. It acts as a bifunctional electrolyte, improving the electrochemical kinetics of both the catholyte and the anolyte. The ionic conductivity of the anolyte and catholyte has been enhanced to 75.2 mS for the catholyte and 3.32 mS for the anolyte by the addition of the ammonium chloride supporting electrolyte. Moreover, the ammonium chloride suppresses dendrite growth in the analyte by forming a complex with the zinc, whereas the Cl^−^ forms a complex with the unstable I_2_ and forms I_2_Cl^−^. These complexes increase iodide utilization by minimizing polyiodide formation and I_2_ precipitation. The cycle life of both sides NH_4_Cl added shows a CE of 93.88% at the 1000^th^ cycle, with VE and EE ranging from 70% to 80%. Also, the KI + NH_4_Cl || ZnCl_2_ + NH_4_Cl cell delivers the improved power density of 74.42 mW cm^−2^ at 114 mA cm^−2^ current density. Therefore, the ammonium chloride electrolyte acts as a bifunctional electrolyte for ZIRBF for commercial‐scale applications.

## 4 Experimental Section

### 4.1 Materials and Methods

Potassium iodide (98%, Sigma–Aldrich, USA), zinc chloride (98%, Sigma–Aldrich, USA), and ammonium chloride (99%, SDFCL) were procured and used without further purification. Graphitic felt with a thickness of 5 mm (SGL Carbon, Germany) was used after the pre‐thermal treatment, and the Nafion‐117 membrane (Fuel Cell Store, USA) was used as received.

### 4.2 Electrolyte Preparation

The catholyte was prepared by dissolving 2 m potassium iodide (KI) in 60 mL of distilled water. At the same time, the anolyte consisted of 2 m zinc chloride (ZnCl_2_) dissolved in 60 mL of distilled water and was prepared and used in the electrochemical analysis. Furthermore, the supporting electrolyte‐based electrolytes were prepared by adding 1 m ammonium chloride (NH_4_Cl) to both the anolyte and catholyte. Here, the non‐metallic and high‐conductivity nature of NH_4_Cl makes it a suitable supporting electrolyte for KI and ZnCl_2_ electrolytes. Very often, ZnI_2_ is used as a catholyte; however, to reduce costs and due to ZnI_2_’s hygroscopic nature, KI is used, which has a higher ionic conductivity than ZnI_2_.

### 4.3 Physical Characterization

Electrolytes and graphite felt were subjected to characterizations such as X‐ray diffraction (XRD) using Bruker D8 with Cu‐K_α_ radiation (𝜆 = 1.5418 Å) at a scan rate of 2 °min^−1^ at a 2θ range of 10°–90°. ATR‐FTIR spectra were recorded using a Bruker model ALPHA II, Germany, at 400–4000 cm^−1^. UV–vis spectroscopy is performed using a Shimadzu instrument over the range 400–800 nm. Using the Field Emission scanning electron microscope (FESEM) model Carl Zeiss SUPRA 55VP, the surface morphology of the felts was depicted.

### 4.4 Electrochemical Studies

Electrochemical characterizations, such as CV and EIS, were performed on the prepared catholyte and anolyte using an OrigaLys electrochemical workstation (France). Both the CV and EIS analyses were carried out using a three‐electrode system, with a graphite felt electrode (1 × 1 cm^2^) as the working electrode, a platinum electrode (1.6 cm^2^) as the counter electrode, and a saturated calomel electrode (SCE _(3 M KCl)_, 0.24 V vs. SHE) as the reference electrode. For catholyte studies, 0.01 m KI was dissolved in 40 mL of deionized (DI) water, while 0.01 m ZnCl_2_ in 40 mL of DI water was used for anolyte studies. Additionally, 1 m NH_4_Cl was added to both solutions for further testing. EIS measurements were carried out over a frequency range of 100 kHz to 100 mHz with an amplitude of 10 mV.

### 4.5 Flow Cell Assembly

The Zn–I_2_ flow cell is constructed using a preheated porous graphite felt as the primary electrode, which features an active area of 2 × 2 cm^2^. The felt electrodes are positioned between two graphite plates, each 6 mm thick, and features 3 mm‐deep grooved channels to facilitate optimal electrolyte flow. To prevent leakage, a 5 × 5 cm^2^ silicon gasket is used between the felt and the graphite plates. To mitigate cross‐contamination, an ion exchange membrane (Nafion‐117) is strategically sandwiched between the plates and the  graphite felt. The electrolytes comprise a catholyte solution of 2 m KI and an anolyte of 2 m ZnCl_2_, with or without 1 m NH_4_Cl, stored in separate reservoirs. These electrolytes are circulated through the graphite felt electrodes via a peristaltic pump, which maintains a consistent flow rate of 70 mL min^−1^. The entire assembly is securely positioned between two copper plates that serve as current collectors. To assess the cell's performance, GCD tests are conducted at various current densities, providing valuable insights into its operational efficiency and effectiveness.

## Author Contributions

**Moothedath Aparnasree**: methodology, data curation, writing – original draft; formal analysis, investigation; **Madeshwaran Mohanraj**: methodology, data curation, writing – original draft; formal analysis; **Anjana Puthanpurayil Jayarajan**: data curation, writing – review, formal analysis, and editing; **Samanth Kokkiligadda**: investigation, formal analysis, writing – review and editing; **Bal Sydulu Singu**: UV–vis and FTIR spectroscopy data curation, formal analysis; **Mani Ulaganathan**: conceptualization, resources, supervision, validation, investigation, project administration, writing – review and editing, **Soong Ho Um**: investigation, resorces, supervision, validation, writing – review and editing.

## Conflicts of Interest

The authors declare no conflicts of interest.

## Supporting information




**Supporting File**: smll74054‐sup‐0001‐SuppMat.docx.

## Data Availability

The data that support the findings of this study are available from the corresponding author upon reasonable request.
