# Supplementary material for: Development of a Stable Electrolyte for Dendrite Suppression and High‐Performance Zn–I2 Redox Flow Battery
Source: Small. 2026 Jun 7;22(43):e13846. doi: 10.1002/smll.202513846 (PMC13432625; doi:10.1002/smll.202513846)
Supplement: Supplementary file 1 — Supporting File: smll74054‐sup‐0001‐SuppMat.docx. [file SMLL-22-e13846-s001.docx]

**SUPPORTING INFORMATION**

**Development of a Stable Electrolyte for Dendrite Suppression and High-Performance Zn–I_2_ Redox Flow Battery**

*Aparnasree Moothedath, Mohanraj Madeshwaran,* *Anjana Puthanpurayil Jayarajan, Bal Sydulu Singu, Ulaganathan* *Mani *,* *Samanth Kokkiligadda*, Soong Ho Um**

M.A.

Department of Chemistry, Amrita School of Physical Sciences Coimbatore, Amrita Vishwa Vidyapeetham, Coimbatore - 641 112, India

M.M., M.U.

Department of Physics, Amrita School of Physical Sciences Coimbatore, Amrita Vishwa Vidyapeetham, Coimbatore - 641 112, India

M.A., M.M., M.U.

Functional Materials Laboratory, Amrita School of Engineering Coimbatore, Amrita Vishwa Vidyapeetham, Coimbatore - 641 112, India

E-mail: ([m_ulaganathan@cb.amrita.edu](mailto:m_ulaganathan@cb.amrita.edu))

B.S.S.

Department of Chemistry, Sreenidhi University, Hyderabad 501301, India

A.P.J., S.K., S.H.U.

School of Chemical Engineering, Sungkyunkwan University, Suwon 16419, Republic of Korea

E-mail: ([samanth213@skku.edu](mailto:samanth213@skku.edu))

S.H.U.

SKKU Advanced Institute of Nanotechnology (SAINT), Sungkyunkwan University, Suwon, Gyeonggi-do, 16419 Korea (S.H.U)

E-mail: ([sh.um@skku.edu](mailto:sh.um@skku.edu))

**Scheme S1:** Schematic representation of ZIRFB

**Table S1**: Diffusion coefficient values of catholyte and anolyte

| **Diffusion coefficient of Catholyte (cm^2^ s^-1^)** | | | | |
| --- | --- | --- | --- | --- |
|  | **KI** | **KI + KCl** | **KI + NH_4_Cl** | **KI + NaCl** |
| Anodic (1) | 2.68*10^-9^ | 15.69*10^-6^ | 29.82*10^-6^ | 12.52*10^-6^ |
| Cathodic (1) | 4.42*10^-9^ | 15.34*10^-6^ | 31.01*10^-6^ | 17.91*10^-6^ |
| Anodic (2) | - | 14.37*10^-6^ | 23.71*10^-6^ | 11.51*10^-6^ |
| Cathodic (2) | - | 20.14*10^-6^ | 53.12*10^-6^ | 20.53*10^-6^ |
| **Diffusion coefficient of Anolyte (cm^2^ s^-1^)** | | | | |
|  | ZnCl_2_ | ZnCl_2_ + KCl | ZnCl_2_ + NH_4_Cl | ZnCl_2_ + NaCl |
| Anodic | 1.61*10^-6^ | 2.44*10^-6^ | 3.21*10^-6^ | 2.11*10^-6^ |
| Cathodic | - | 2.21*10^-6^ | 2.17*10^-6^ | 1.74*10^-6^ |

**Table S2**: Comparison of solution resistance, charge transfer resistance, exchange current density and rate constant of catholytes.

| **Electrolyte** | **Solution resistance R_s_(Ω)** | **Charge transfer resistance R_ct_(Ω)** | **Exchange current density (i_0_)**  **(A cm^-2^)** | **Rate constant (K)**  **(cm s^-1^)** |
| --- | --- | --- | --- | --- |
| Bare_KI | 90.428 | 6.03 | 4.257*10^-3^ | 4.413*10^-6^ |
| KI + KCl | 1.77 | 5.31 | 4.844*10^-3^ | 5.019*10^-6^ |
| KI+NH_4_Cl | 1.28 | 4.81 | 5.337*10^-3^ | 5.530*10^-6^ |
| KI + NaCl | 16.48 | 5.22 | 4.91*10^-3^ | 5.096*10^-6^ |

**Equation S1**

**Exchange current density** ${\boldsymbol{(}\boldsymbol{i}}_{\boldsymbol{0}}$**):**

$$Exchange current density \left( i_{0} \right)=\frac{RT}{nFR_{ct}}----------(a)$$

**Rate constant (K):**

$$Rate constant\left( K \right)=\frac{i}{nFC} ----------(b)$$

Where n- number of electron transfer, F- faraday constant, T temperature in kelvin, R- Gas constant and C- concentration.

**Figure S2**: Real image of the constructed ZIRFB

**
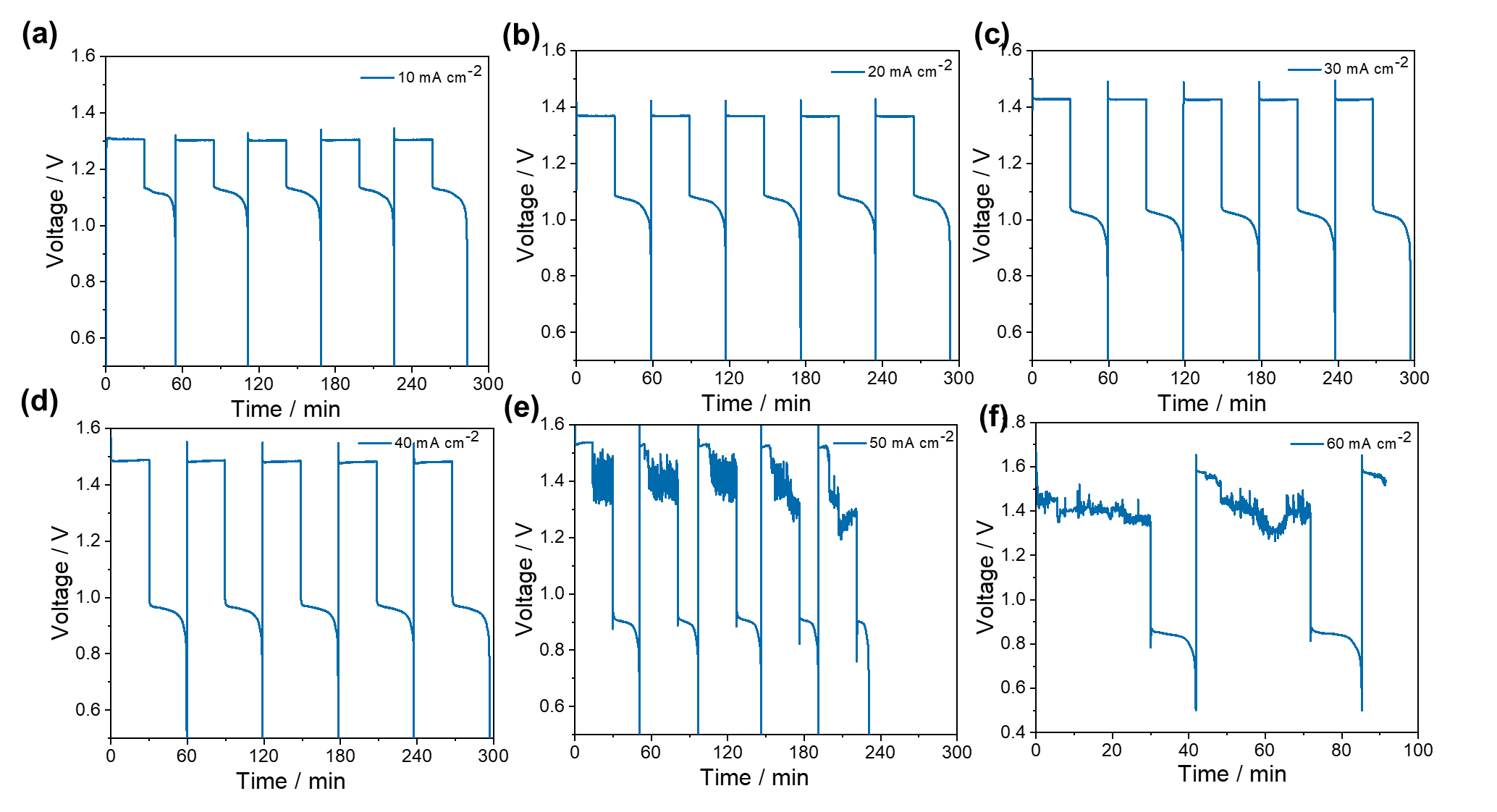
**

**Figure S3**: GCD profile of bare KI || ZnCl_2_ at different current densities with 30 minutes charge-discharge duration: (a) At 10 mA cm^-2^; (b) 20 mA cm^-2^; (c) 30 mA cm^-2^; (d) 40 mA cm^-2^; (e) 50 mA cm^-2^;(f) 60 mA cm^-2^.


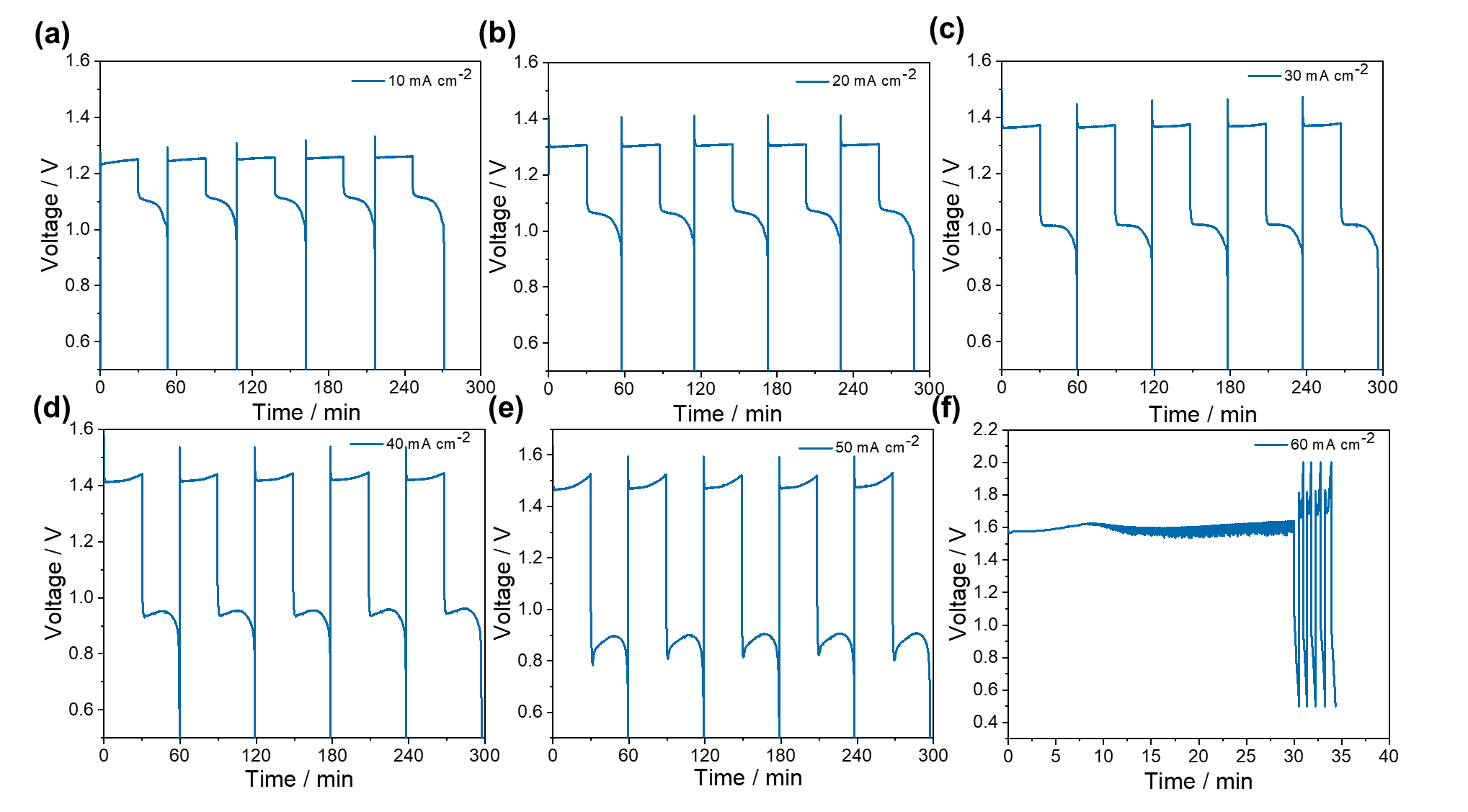


**Figure S4**: GCD profile of bare KI_NH_4_Cl|| ZnCl_2_ at different current densities with 30 minutes charge-discharge duration: (a) At 10 mA cm^-2^; (b) 20 mA cm^-2^; (c) 30 mA cm^-2^; (d) 40 mA cm^-2^; (e) 50 mA cm^-2^;(f) 60 mA cm^-2^.


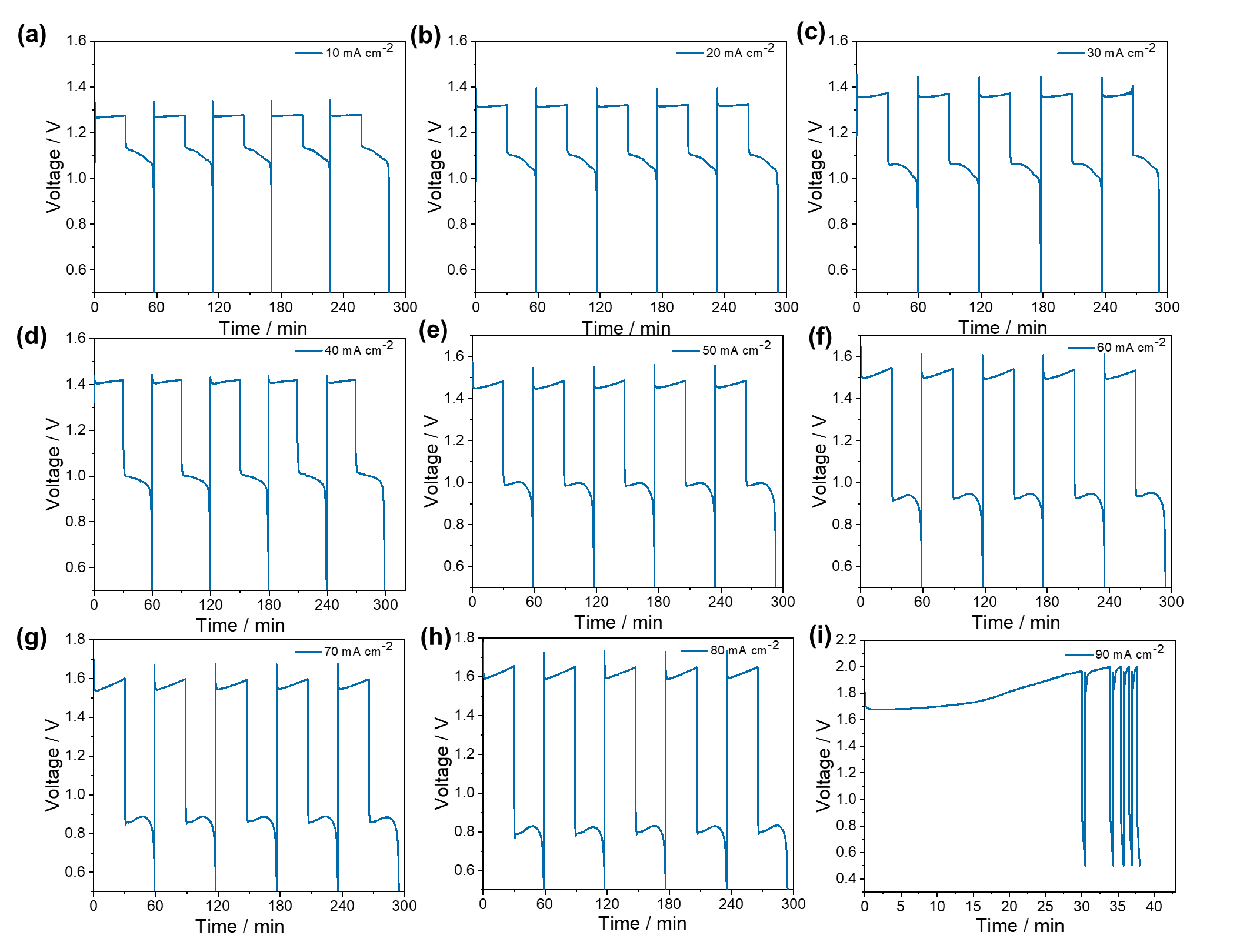


**Figure S5**: GCD profile of bare KI_NH_4_Cl|| ZnCl_2__NH_4_Cl at different current densities with 30 minutes charge-discharge duration: (a) At 10 mA cm^-2^; (b) 20 mA cm^-2^; (c) 30 mA cm^-2^; (d) 40 mA cm^-2^; (e) 50 mA cm^-2^;(f) 60 mA cm^-2^; (g) 70 mA cm^-2^; (h) 80 mA cm^-2^; (f) 90 mA cm^-2^.

**Equation S2:**

**Coulomb efficiency (CE)** is the percentage ratio of the charge delivered from the electrode during discharging (Q dis) to the charge stored on the electrode during charging (Q cha), which is related to the rapid reaction rate.

$$CE=\frac{Discharge capacity}{Charge capacity} \times100 \%----------(a)$$

Where capacity of a cell can be calculated by the equation:

$Capacity=I\times t$

Where, I is the current passed, t- charge and discharge time

**Voltage efficiency (VE)** is a measure of how well a device or battery performs about its voltage. Voltage efficiency is the ratio of the working voltage (discharge) to the charge voltage. It's also known as voltaic efficiency, which is the ratio of the average discharge voltage to the average charge voltage.

$$VE= \frac{Discharge voltage}{Charge voltage} \times100 \%----------(b)$$

**Energy efficiency (EE)** is a measure for the amount of energy that can be taken from the battery compared to the amount of energy that was charged into the battery beforehand.

$$EE=\frac{CE\times VE}{100} \%----------(c)$$


**Figure S6**: (a) Efficiency comparison of bare KI || ZnCl_2_, KI + NH_4_Cl || ZnCl_2_, KI + NH_4_Cl || ZnCl_2_ + NH_4_Cl at 40 mA cm^-2^; (b) Current density vs Specific capacity graph of KI + NH_4_Cl || ZnCl_2_ + NH_4_Cl; (c) Comparison of efficiencies at 150^th^ cycle for all the three cells.

**Equation S3:**

$$Cell capacity \left( C \right)= \frac{I\times t}{3.6}(Ah)----------(a)$$

$$Specific capcity \left( C_{sp} \right)= \frac{I\times t}{V\times3.6}(Ah L^{-1})----------(b)$$

$$Energy density \left( ED \right)= C\times V(Wh L^{-1}) ----------(c)$$

$$Power density \left( PD \right)= j\times V (W {cm}^{-2}) ----------(d)$$

Where I is the current (A), t is the discharge time (h), V is the volume (L), C capacity (Ah), j current density (A cm^-2^), V voltage of the cell

**Figure S7:** FTIR analysis of the cycled anolytes: (a) ZnCl_2_ after 160 cycles; (b) ZnCl_2_+NH_4_Cl after 1000 cycles.

**Figure S8**: Image of the anode felt after cycle life: (a) KI || ZnCl_2_ after 160^th^ cycle; (b) KI_NH_4_Cl || ZnCl_2_ after 950^th^ cycle; (c) KI_NH_4_Cl || ZnCl_2__NH_4_Cl after 1000^th^ cycle.


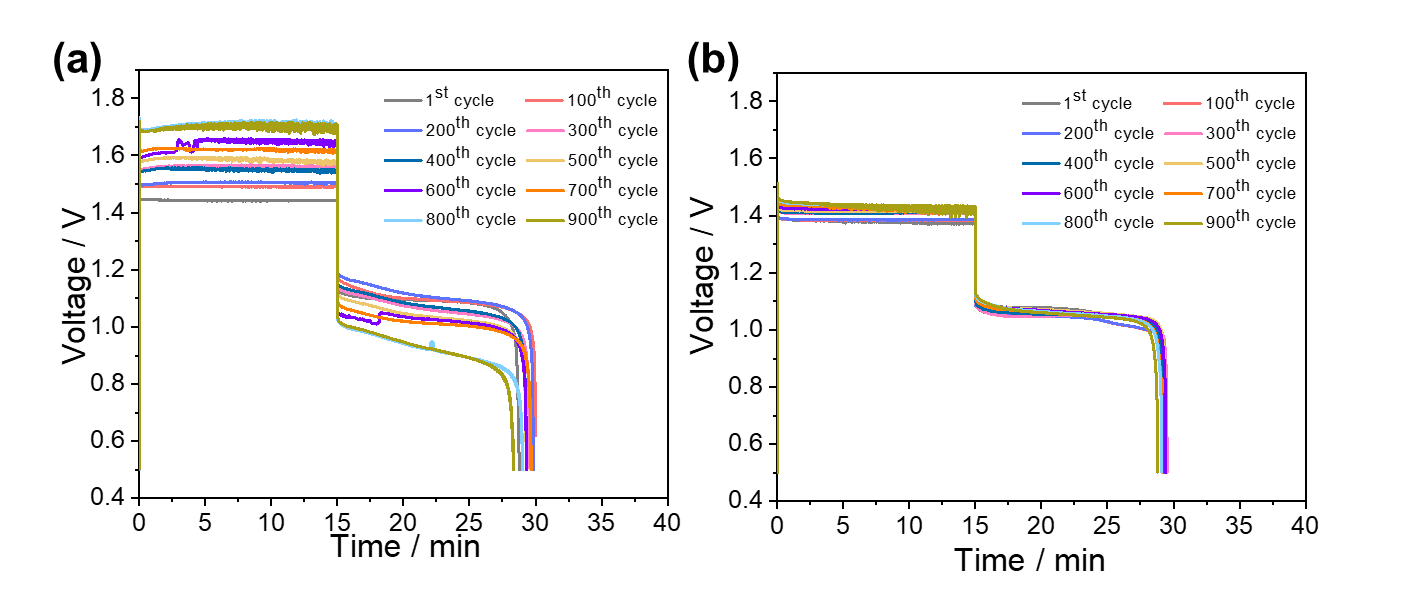


**Figure S9**: Cycle life profile of each 100^th^ cycle; (a) KI + NH_4_Cl || ZnCl_2_; (b) KI + NH_4_Cl || ZnCl_2_+ NH_4_Cl.

**Table S3**: EIS data comparison of the constructed flow cell

| **Flow cells** | **Solution resistance R_s_(Ω)** | **Charge transfer resistance R_ct_(Ω)** | **Exchange current density (i_0_)**  **(A cm^-2^)** | **Rate constant (K)**  **(cm s^-1^)** |
| --- | --- | --- | --- | --- |
| KI \|\| ZnCl_2_ | 1.521 | 3.418 | 3.75*10^-3^ | 9.71*10^-6^ |
| KI+NH_4_Cl \|\| ZnCl_2_ | 1.504 | 3.212 | 3.99*10^-3^ | 10.31*10^-6^ |
| KI+NH_4_Cl \|\| ZnCl_2_+NH_4_Cl | 1.451 | 2.768 | 4.637*10^-3^ | 12.01*10^-6^ |

**Table S4**: Zinc-Iodide Redox flow battery comparison

| **SI.**  **no** | **Electrode**  **Area (cm^2^)** | **Catholyte (M)** | **Anolyte (M)** | **Supporting electrolyte (M)** | **Additives** | **Cycle number @ j (mA cm^-2^)** | **(CE,VE,EE)%** | **Flow rate**  **(mL/min)** | **Membrane** | **Power density**  **(mW cm^-2^ @ mA cm^-2^)** | **Ref** |
| --- | --- | --- | --- | --- | --- | --- | --- | --- | --- | --- | --- |
| 1. | GF, 9 | 2.5 NH_4_I | 1.25 ZnCl_2_ | 1.25 NH_4_Cl | - | 1200 @ 20 | (99,89,88) | 1 | Nafion-115 | 75 @ 100 | ^[1]^ |
| 2. | CF, 28 | 6 KI | 3 ZnCl_2_ | KCl | 5 wt % PVP | 600 @ 20 | (-,78,70) | 39 | Perfluorinated sulfonic acid | - | ^[2]^ |
| 3. | GF | 1 ZnI_2_ | 1 ZnI_2_ | - | 1 M NH_4_Br | 100 @ 40 | (-, -,85) | 40 | Nafion-211,115 | 310 @ 480 | ^[3]^ |
| 4. | GF, 2 | 1 M ZnI_2_ | 1 ZnI_2_ | - | 7.5 wt% CAN | 170 @ 100 | (96,61.5,60) | 47 | Nafion-115 | - | ^[4]^ |
| 5. | GF, 40 | 3.5 ZnI_2_ | 2.5 ZnI_2_ | - |  | 40 @ 10 | - | 50 | Nafion-115 | - | ^[5]^ |
| 6. | CF, 13.5 | 6 KI | 3 ZnBr_2_ | 3 NH_4_Br, 2 KBr | 3 M KSCN | 202 @ 20 | (99.6, -,56.34) | 30 | Nafion -212 | 206.3 @ 302.21 | ^[6]^ |
| 7. | GF  (Ti-mesh) | 1.5 NaI | 0.5 ZnCl_2_ | - | 20 wt% PC | 50 @ 20 | (90, -, -) | 200 | Durapor-membarne | - | ^[7]^ |
| 8. | GF, 48 | 2 KI | ZnBr_2_ | 2 KCl | ZnBr_2_ | 300 @ 80 | (96, -,82) | 50 | Polyolefin porous | - | ^[8]^ |
| 9. | GF, 4 | 5 ZnI_2_ | 5 ZnI_2_ | - | 2.5 M ZnBr_2_ | 20 @ 10 | (95, -, -) | 10 | Nafion-117,115 | 50 @ 70 | ^[9]^ |
| 10. | GF, 4 | 2 KI | 2 ZnCl_2_ | 1 NH_4_Cl | - | 1000 @ 30 | (93.88,75.12,70.52) | 70 | Nafion-117 | 74.8 @ 114 | This work |

**Reference**

[1] M. Mousavi, G. Jiang, J. Zhang, A. G. Kashkooli, H. Dou, C. J. Silva, Z. P. Cano, Y. Niu, A. Yu, Z. Chen, *Energy Storage Mater* **2020**, *32*, 465.

[2] J. Yang, Y. Song, Q. Liu, A. Tang, *J Mater Chem A Mater* **2021**, *9*, 16093.

[3] Q. P. Jian, M. C. Wu, H. R. Jiang, Y. K. Lin, T. S. Zhao, *J Power Sources* **2021**, *484*, 229238.

[4] Y. Zhao, Y. Li, J. Mao, Z. Yi, N. Mubarak, Y. Zheng, J.-K. Kim, Q. Chen, *J Mater Chem A Mater* **2022**, *10*, 14090.

[5] B. Li, Z. Nie, M. Vijayakumar, G. Li, J. Liu, V. Sprenkle, W. Wang, *Nat Commun* **2015**, *6*, 6303.

[6] B. Lu, M. Yang, M. Ding, S. Yan, W. Xiang, Y. Cheng, H. Fu, Z. Xu, C. Jia, *SusMat* **2023**, *3*, 522.

[7] S. Ito, M. Sugimasa, Y. Toshimitsu, A. Orita, M. Kitagawa, M. Sakai, *Electrochim Acta* **2019**, *319*, 164.

[8] C. Xie, H. Zhang, W. Xu, W. Wang, X. Li, *Angewandte Chemie International Edition* **2018**, *57*, 11171.

[9] G.-M. Weng, Z. Li, G. Cong, Y. Zhou, Y.-C. Lu, *Energy Environ Sci* **2017**, *10*, 735.
